# Peptidoglycan binding by a pocket on the accessory NTF2-domain of Pgp2 directs helical cell shape of *Campylobacter jejuni*

**DOI:** 10.1016/j.jbc.2021.100528

**Published:** 2021-03-10

**Authors:** Chang Sheng-Huei Lin, Anson C.K. Chan, Jenny Vermeulen, Jacob Brockerman, Arvind S. Soni, Martin E. Tanner, Erin C. Gaynor, Lawrence P. McIntosh, Jean-Pierre Simorre, Michael E.P. Murphy

**Affiliations:** 1 Department of Microbiology and Immunology, University of British Columbia, Vancouver, British Columbia, Canada; 2 Department of Biochemistry and Molecular Biology, University of British Columbia, Vancouver, British Columbia, Canada; 3 Department of Chemistry, University of British Columbia, Vancouver, British Columbia, Canada; 4 University of Grenoble Alpes, CNRS, CEA, IBS, Grenoble, France

**Keywords:** *Campylobacter*, carboxypeptidase, peptidoglycan, crystal structure, nuclear magnetic resonance (NMR), docking, bacterial cell shape, NTF2 domain, AIR, ambiguous interaction restraint, CSP, chemical shift perturbation, HPLC, high performance liquid chromatography, PG, peptidoglycan

## Abstract

The helical morphology of *Campylobacter jejuni*, a bacterium involved in host gut colonization and pathogenesis in humans, is determined by the structure of the peptidoglycan (PG) layer. This structure is dictated by trimming of peptide stems by the LD-carboxypeptidase Pgp2 within the periplasm. The interaction interface between Pgp2 and PG to select sites for peptide trimming is unknown. We determined a 1.6 Å resolution crystal structure of Pgp2, which contains a conserved LD-carboxypeptidase domain and a previously uncharacterized domain with an NTF2-like fold (NTF2). We identified a pocket in the NTF2 domain formed by conserved residues and located ∼40 Å from the LD-carboxypeptidase active site. Expression of *pgp2* in trans with substitutions of charged (Lys257, Lys307, Glu324) and hydrophobic residues (Phe242 and Tyr233) within the pocket did not restore helical morphology to a *pgp2* deletion strain. Muropeptide analysis indicated a decrease of murotripeptides in the deletion strain expressing these mutants, suggesting reduced Pgp2 catalytic activity. Pgp2 but not the K307A mutant was pulled down by *C. jejuni* Δ*pgp2* PG sacculi, supporting a role for the pocket in PG binding. NMR spectroscopy was used to define the interaction interfaces of Pgp2 with several PG fragments, which bound to the active site within the LD-carboxypeptidase domain and the pocket of the NTF2 domain. We propose a model for Pgp2 binding to PG strands involving both the LD-carboxypeptidase domain and the accessory NTF2 domain to induce a helical cell shape.

*Campylobacter jejuni* is a Gram-negative, highly motile and helical-shaped bacterium that is a leading cause of bacterial foodborne gastroenteritis worldwide (1). This pathogen is commonly found as a commensal colonizer of livestock, especially poultry (2). Consumption of contaminated food, water or contact with domesticated animals can lead to human infection. Disease symptoms vary from self-limiting diarrhea to serious sequelae including inflammatory bowel disease and Guillain–Barré syndrome. Treatment of *C. jejuni* infection is challenged by the emergence of antibiotic-resistant strains (3).

Intestinal colonization is the first step in *C. jejuni* infection. The viscous mucus layer forms a physical barrier to prevent bacterial invasion. Flagellar motility is required for *C. jejuni* colonization, as only motile strains were isolated from human volunteers challenged with a mixture of flagellated and aflagellated bacteria (4, 5). Two rod-shaped mutant *C. jejuni* strains (Δ*pgp1* and Δ*pgp2*) with no discernable defects in cell growth, stress survival, nor swimming velocity in nonviscous liquid media show a 20–40% decrease in motility as compared with wild-type helical cells in semisolid agar (6, 7). Moreover, both strains are severely impaired in passaging through the mucus layer to reach the intestinal epithelium in a mouse model (8). Finally, these mutants exhibit a 2–3 log decrease in the chick colonization model as compared with wild-type (6, 7), highlighting the importance of helical shape in host colonization.

Peptidoglycan (PG) is the major cell shape determinant, as the purified polymer sacculus retains the original cell morphology when isolated (9). *C. jejuni* PG is synthesized from alternating *N*-acetylglucosamine (GlcNAc) and *N*-acetylmuramic acid (MurNAc) saccharides with a pentapeptide (L-Ala^1^-γ-D-Glu^2^-*m*-DAP^3^-D-Ala^4^-D-Ala^5^) attached to the MurNAc moiety (10). Nascent PG strands are incorporated into the sacculus and are cross-linked at the D-Ala^4^➝(D) *m*-Dap^3^ (4-3) positions on the peptide stems by transpeptidases. PG hydrolases can cleave cross-linkages (11), trim peptides (6, 7, 11), and remove the O-acetyl group from MurNAc (12). These modifications are required for generating the helical shape of *C. jejuni*. Mutant strains with nonhelical shapes such as straight-rod or highly curved showed altered muropeptide profiles (6, 7, 12).

Pgp2 is an LD-carboxypeptidase that cleaves D-Ala from both monomeric and cross-linked tetrapeptides (7). Tripeptides were not detected in an analysis of a *pgp2* deletion strain of *C. jejuni*, which displayed a straight-rod morphology (7). Rod-shaped isolates from a transposon library carried mutations within *pgp2* or a second hydrolase-encoding gene, *pgp1* (13). The tripeptide product of Pgp2 cleavage is the substrate for Pgp1, and a Δ*pgp1pgp2* strain shares a similar muropeptide profile to that of the Δ*pgp2* strain (7). Homologs of Pgp1 and Pgp2 are characterized in *Helicobacter pylori*, and both are required for maintaining helical cell shape (14, 15). The *H. pylori* homolog of Pgp2 is named Csd6 and shares 36% amino acid sequence identity.

Sequence analysis of Pgp2 indicates that it contains an LD-carboxypeptidase (LD-CPase) domain and an NTF2-like superfamily (NTF2) domain. NTF2 domains are broadly found in nature and function in both ligand binding and catalysis, including in other PG hydrolases such as PBP2a and NlpC/p60 (16, 17). Csd6 is suggested to be required for flagellin biosynthesis in *H. pylori* (18), and its NTF2 domain is proposed to bind a pseudaminic acid (19).

Here, we investigated the role of the NTF2 domain in maintaining *C. jejuni* helical shape. We report the crystal structure of Pgp2 and identified a conserved binding pocket in the NTF2 domain. Site-directed mutagenesis combined with interaction studies using PG fragments was used to show that the NTF2 domain binds PG and is required for *C. jejuni* helical shape. On the basis of these results, we propose a model for Pgp2-PG interaction involving both the LD-CPase and the NTF2 domains to guide catalytic activity.

## Results

### Two clusters of conserved residues are identified in the Pgp2 structure

**Figure 1:**
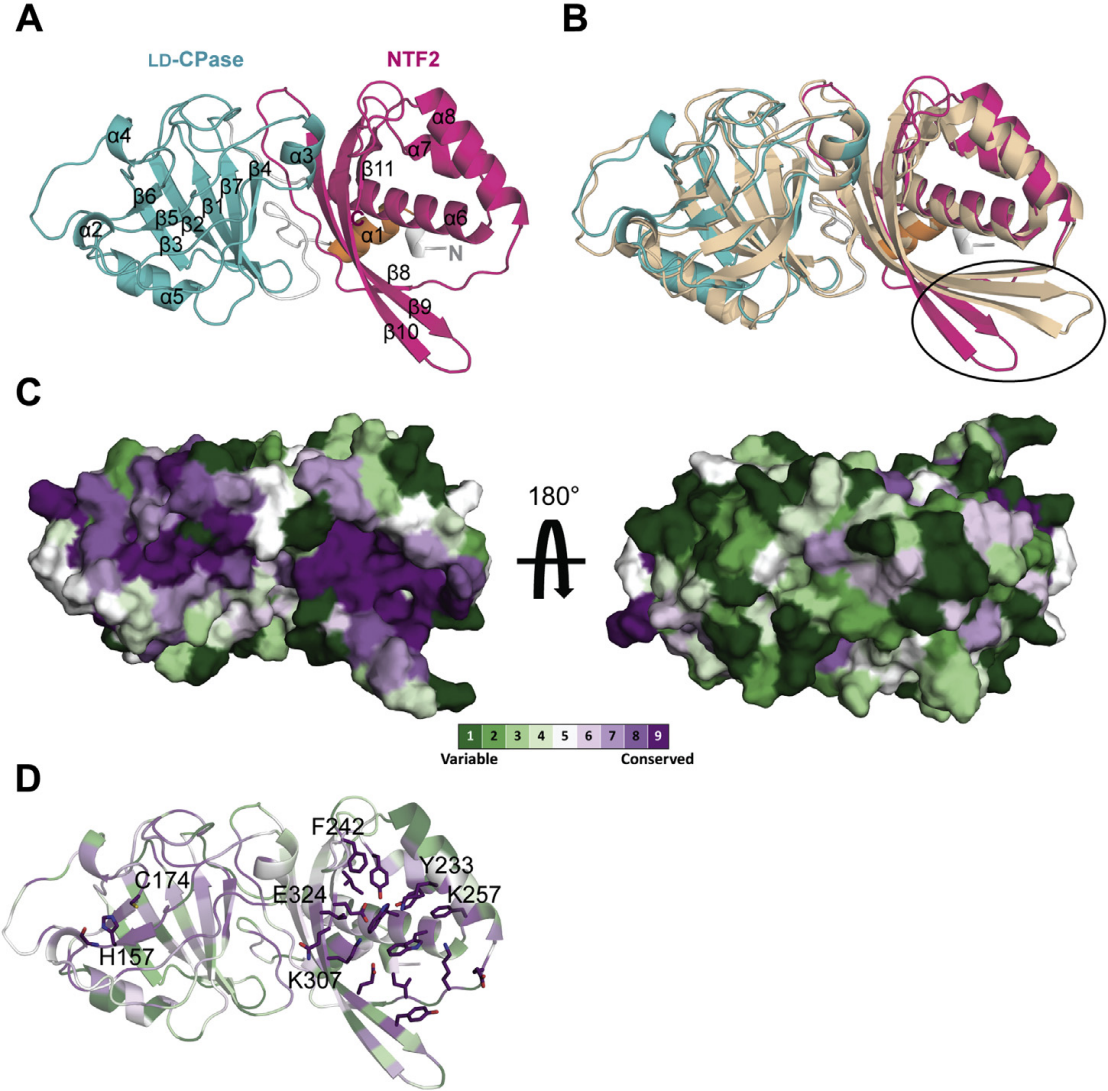
**The conserved residues of Pgp2 are focused in two clusters: the active site of the LD-CPase domain and the pocket of NTF2 domain**. *A*, The overall monomeric structure of Pgp2. The N-terminal helix, LD-CPase and NTF2 domains, and loops between domains are colored *orange*, *cyan*, *magenta*, and *gray*, respectively. *B*, superimposition of Pgp2 and *H. pylori* Csd6 (*orange*, PDB ID: 4XZZ). The strands β9–β10 of Pgp2 (*circled*) showed the largest deviation from the Csd6 model. *C*, surface representation of Pgp2 colored by amino acid conservation. The most conserved residues are shown in *purple* and the least conserved in *green*. *D*, the clustered conserved residues are shown in stick form (nitrogen, *blue*; oxygen, *red*; sulfur, *yellow*). Residues targeted for subsequent site-directed mutagenesis studies are labeled.

A recombinant Pgp2 construct (Pgp2^43-325^) containing residues 43–325 of the native sequence (*cjj81176_0915*) is enzymatically active (20) and was suitable for structural characterization. Pgp2 crystallized in space group *P*2_1_2_1_2_1_ and the structure was solved to 1.6 Å resolution by molecular replacement using Csd6 as a search model (PDB ID: 4XZZ). X-ray data collection and refinement statistics are summarized in Table S1. The Pgp2 structure contains one Pgp2 molecule in the asymmetric unit (Fig. 1*A*). Analysis in solution by dynamic light scattering indicated an average molecular weight of 44 kDa, consistent with the predicted weight of the recombinant Pgp2 monomer (36 kDa; not shown).

The structure of Pgp2 contains an N-terminal helix (residues Q43-I51), the catalytic LD-CPase domain (residues V65-K201), and the C-terminal NTF2 domain (residues K208-Q325), each connected to the next domain by a single loop (Fig. 1*A*). An extensive interface with a buried surface area of 1130 Å^2^ is found between the LD-CPase and NTF2 domains, as calculated by the PISA server (21). The LD-CPase domain consists of parallel strands β5–β6 sandwiched by a beta sheet (β1–β4 and β7), four helices (α2–α5), and connecting loops. The likely catalytic triad (C174-H157-G158) is located on the central stands β5–β6 (Fig. 1, *A* and *D*). The NTF2 domain contains three helices (α6–α8) wrapped around a curved antiparallel β-sheet (β8–β11), which together forms a cone shape domain with a broad pocket.

Using a Dali search (22) for similar structures in the PDB, only Csd6 can be superimposed with Pgp2 over both the LD-CPase and NTF2 domains (PDB ID: 4XZZ, RMSD of 1.8 Å for 281 Cα atoms). The structure of Csd6 includes two additional α-helices at the N terminus that forms a homodimerization domain between two Csd6 monomers. The equivalent 27 residues are absent in the truncated Pgp2 construct. The structure of Csd6 deviates from strands β9–β10 of the Pgp2 NTF2 domain (Fig. 1*B*). The LD-CPase domain of Pgp2 is distantly related to LD-transpeptidases with sequence identity <20%. An example is LdtMt5 from *Mycobacterium tuberculosis* (PDB ID: 4Z7A, RMSD of 2.4 Å over 106 Cα atoms). The NTF2 domain is structurally similar to proteins with diverse functions, such as calmodulin-dependent protein kinase II from *Rattus norvegicus* (PDB ID: 5U6Y, RMSD of 2.4 Å over 109 Cα atoms) and penicillin-binding protein 2A, a DD-transpeptidase from methicillin-resistant *Staphylococcus aureus* (PDB ID: 3ZFZ, RMSD of 2.7 Å over 95 Cα atoms).

To predict functionally important residues in Pgp2, 150 homologous sequences with 35%–95% sequence identity to Pgp2 were identified and aligned in ConSurf (23). The level of sequence conservation was mapped onto the surface of Pgp2 (Fig. 1*C*). Regions of high sequence conservation are observed on one side of the molecule, focused in two clusters. The first cluster is in proximity to the catalytic triad in the LD-CPase domain (Fig. 1*D*). The second cluster is formed by 17 primarily aromatic (41%) and charged (30%) residues that are clustered in the pocket of the NTF2 domain (Fig. 1*D*).

### The LD-CPase and NTF2 domains are required for helical shape

**Figure 2:**
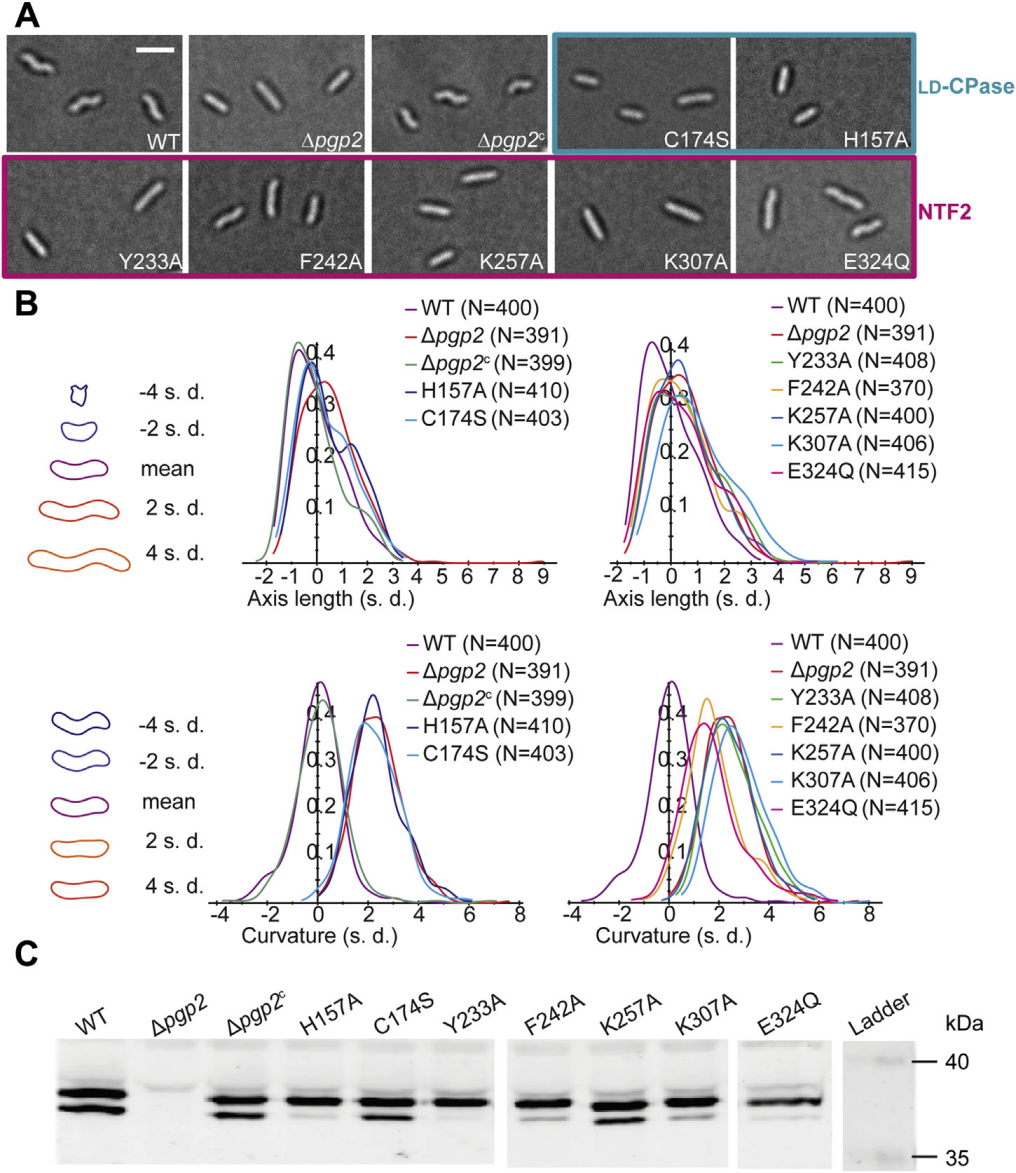
**Helical-shape restoration of Δ*pgp2* by complementation with wild-type *pgp2* and point mutants of the catalytic triad and within the NTF2 pocket.***A*, light microscope images of *C. jejuni* strains. Scale bar = 2 μm. *B*, quantitative analysis of the shape of individual cells extracted from images using Celltool software. The shape mode that best describes the shape variance in each analysis (axis length or curvature) is plotted on the left side. Smooth histograms display the population of cells by axis length or curvature (*x*-axis). *C*, composite Western blot of *C. jejuni* whole cell extracts for Pgp2. Samples were normalized to total protein using a Bradford assay. Spliced lanes are separated by white lines. See Figure S1 for images of the full blots.

Previously, integration of wild-type *pgp2* with its native promotor at a remote site of the Δ*pgp2* chromosome restored helical cell shape in the straight-rod deletion strain (7). Using this complementation system, we evaluated the importance of the two conserved clusters by constructing single residue Pgp2 variants. Mutants of *pgp2* encoding substitutions of catalytic residues (H157 and C174) or residues in the NTF2 pocket (Y233, F242, K257, K307, and E324) were generated, and the morphologies of these strains were examined by differential interference contrast microscopy. The Δ*pgp2* strains complemented by mutants of the catalytic triad (H157A, C174S) displayed rod morphologies, confirming that Pgp2 catalytic activity is required for helical shape (Fig. 2*A*). Point mutations within the NTF2 pocket resulted in bacteria with partially curved to straight morphologies. Quantitative analysis of each strain by Celltool (24) indicated that strains complementation with native *pgp2* displayed a cell curvature distribution similar to wild-type cells, whereas the strains expressing Pgp2 mutants had similar lengths but varied in cell curvature (Fig. 2*B*). The catalytic triad mutants (C174S, H157A) and three mutants within the NTF2 pocket (Y233A, K257A, and K307A) resulted in bacteria with cell curvature distributions that were similar to the Δ*pgp2* strain. Two mutants were of an intermediate phenotype (F242A and E324Q). These curvature defects were not due to Pgp2 expression deficiency as confirmed by Western blot using an anti-Pgp2 antibody (Figs. 2*C* and S1). Together, we concluded that the LD-CPase and NTF2 domains are both required for helical shape in *C. jejuni*.

Pgp2 was reported to be post-translationally modified (25). In this earlier study, Pgp2 purified from cell lysate using a carbohydrate-binding column displayed three spots by two-dimensional electrophoresis. Four *N*-linked glycosylation sequons (Asn-Xaa-Ser/Thr) were found in the sequence of Pgp2 suggesting that post-translational glycosylation likely gave rise to the two bands observed on the Western blot (Fig. 2*C*). No positive correlation was found from a quantification of the intensities of the two bands and a comparison against cell shape.

### The NTF2 domain is required for Pgp2 catalytic activity in *C. jejuni*

**Figure 3:**
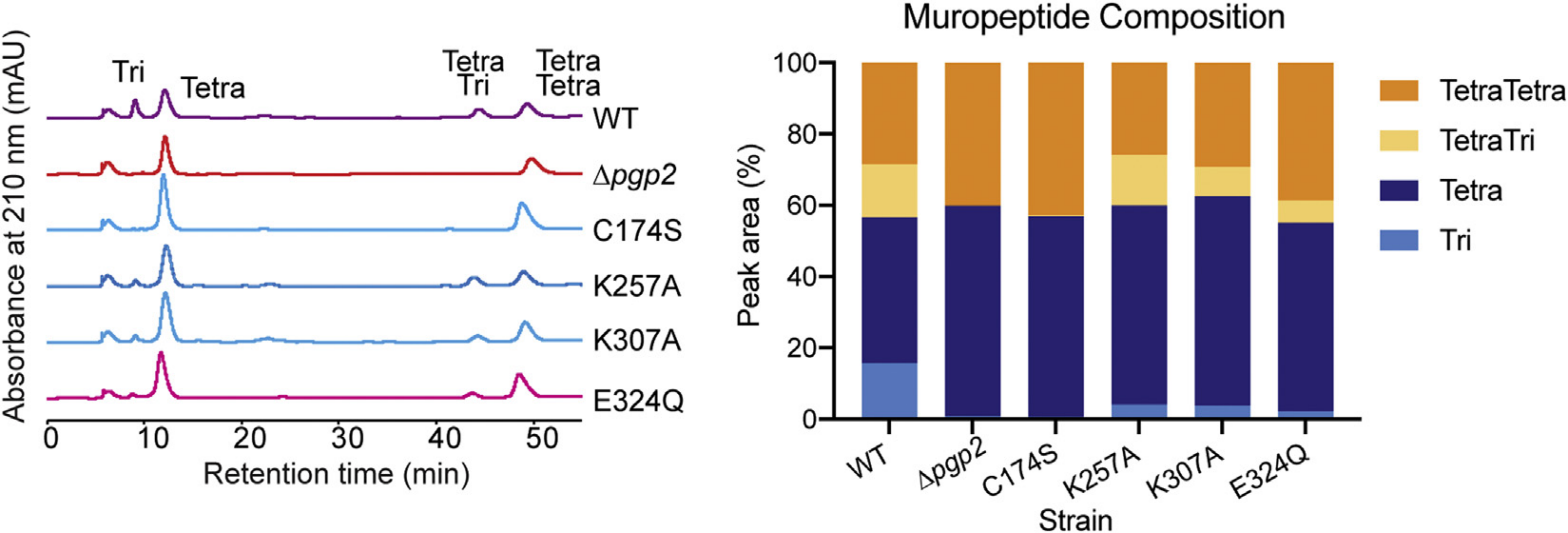
**HPLC muropeptide profile of *C. jejuni* wild-type, Δ*pgp2* and Δ*pgp2* complemented with point mutations of the catalytic triad and within the NTF2 pocket.** Purified PG digested with muramidase was reduced with sodium borohydride and separated by HPLC. Muropeptides were monitored by absorbance at 210 nm. Each peak is labeled with the corresponding muropeptide. The proportion of each muropeptide (Tri, Tetra, TetraTri, or TetraTetra), relative to the combined total of these four muropeptides, is shown on the right.

Pgp2 activity was quantified by assaying for the products (tripeptide or cross-linked tetratripeptides) in the hydrolyzed PG of *C. jejuni*. Purified PG from *C. jejuni* strains complemented with *pgp2* harboring point mutations was digested by muramidase and analyzed by high performance liquid chromatography (HPLC). The identities of peaks from the HPLC elution profile were confirmed by matrix-assisted laser desorption ionization-time of flight mass spectrometry. Complementation with a catalytically inactive variant (C174S) produced undetectable levels of monomeric and cross-linked tripeptides (Fig. 3, left panel), and this variant served as a negative control. Lower levels of products were observed in the NTF2 domain mutant strains (K257A, K307A, and E324Q) relative to the strain expressing wild-type Pgp2 (Fig. 3, left panel). The proportion of monomeric tripeptides ranged from 14% to 25%, whereas the proportion of cross-linked tetratripeptides ranged from 41% to 95% (Fig. 3, right panel), demonstrating that the NTF2 domain is required for full Pgp2 activity in *C. jejuni.* Furthermore, for NTF2 mutants, the activity on monomeric tetrapeptides was diminished more than for cross-linked tetrapeptides.

### The NTF2 domain binds PG

In some multidomain PG hydrolases, the presence of a PG-binding domain enhances the activity of the catalytic domain (26, 27). We examined whether the NTF2 domain functions as a PG-binding domain that is synergistic with the LD-CPase domain for Pgp2 catalytic activity. A pull-down experiment was performed with Pgp2^43-325^ and variants using Δ*pgp2* PG, which is rich in tetrapeptides. Wild-type Pgp2 was pulled down, demonstrating PG binding (Fig. S2*A*). Two catalytically inactive variants, C174S (Pgp2^C174S^) and H157A (Pgp2^H157A^), showed minimal association. The K307A variant (Pgp2^K307A^), a conserved residue within the pocket of the NTF2 domain, was also weakly associated, supporting a role for this binding pocket for appreciable PG interaction. Mutation of conserved Y233 in the NTF2 domain (Pgp2^Y233F^) was insufficient to disrupt the pull down of Pgp2 by PG. The removal of one hydroxyl group by replacement of tyrosine by phenylalanine was insufficient to substantively decrease PG binding.

### Strands β9–β10 form a flexible lip of the NTF2 domain binding pocket

To investigate if the loss of PG binding by the Pgp2^K307A^ was due to conformational change, the crystal structure of this variant was solved at 1.85 Å resolution (Table S1). The Pgp2^K307A^ crystal structure contains two Pgp2^K307A^ molecules in one asymmetric unit that superimposed with an RMSD of 0.7 Å over 281 Cα atoms. The fold of the Pgp2^K307A^ is similar to that of wild-type Pgp2 (RMSD of 1.5 Å over 277 aligned Cα atoms). The largest deviation is located at residues 296–306 of strands β9–β10 (Fig. S3, *A*–*C*), which form a protruding lower lip of the NTF2 domain binding pocket. An overlay of the structures of Pgp2, Pgp2^K307A^, and *H. pylori* Csd6 (PDB ID: 4XZZ) showed that the conformation of lower lip is more similar between the latter two (Fig. S3, *A*–*D*). In the wild-type Pgp2 crystal structure, the average B-factor of β9-β10 (residues 292–310) was 53.4 Å^2^, 1.8-fold higher than the average B-factor over all residues. We conclude that β9–β10 is conformationally flexible and may allow variation in ligand specificity.

### Identifying PG-binding interfaces of Pgp2 by NMR-monitored titrations

To identify PG interaction sites by NMR spectroscopy, triple-labeled (^2^H-^13^ C-^15^N) Pgp2^43-325^ was produced and used to obtain the assignments of 236 out of 287 expected main chain amide ^1^H^N^-^15^N signals (Fig. S4*A*). The unassigned residues include those in two loops (residues 167–173 and 238–253) for which conformational exchange may have led to resonance broadening. Although deuteration was required for resonance assignments of this 36 kDa protein, the ^15^N-BEST-TROSY-HSQC spectrum of ^15^N-labeled Pgp2^43-325^ was of high quality (Fig. S4B), enabling titration experiments with panel of PG-derived ligands.

Four PG ligand preparations for titration experiments were derived from digesting whole PG: a muramidase digestion of *C. jejuni* Δ*pgp2* PG, a DL-endopeptidase (*Pseudomonas aeruginosa* Tse1) digestion of *Escherichia coli* PG, HPLC-purified murotetrapeptide, and HPLC-purified cross-linked murotetrapeptides. The latter two ligands were treated with sodium borohydride before purification, which reduced the MurNAc residue. A fifth ligand was a synthesized peptide analogue (D-Glu-*m*-oxa-Dap-D-Ala) of the Pgp2 substrate (20).

**Figure 4:**
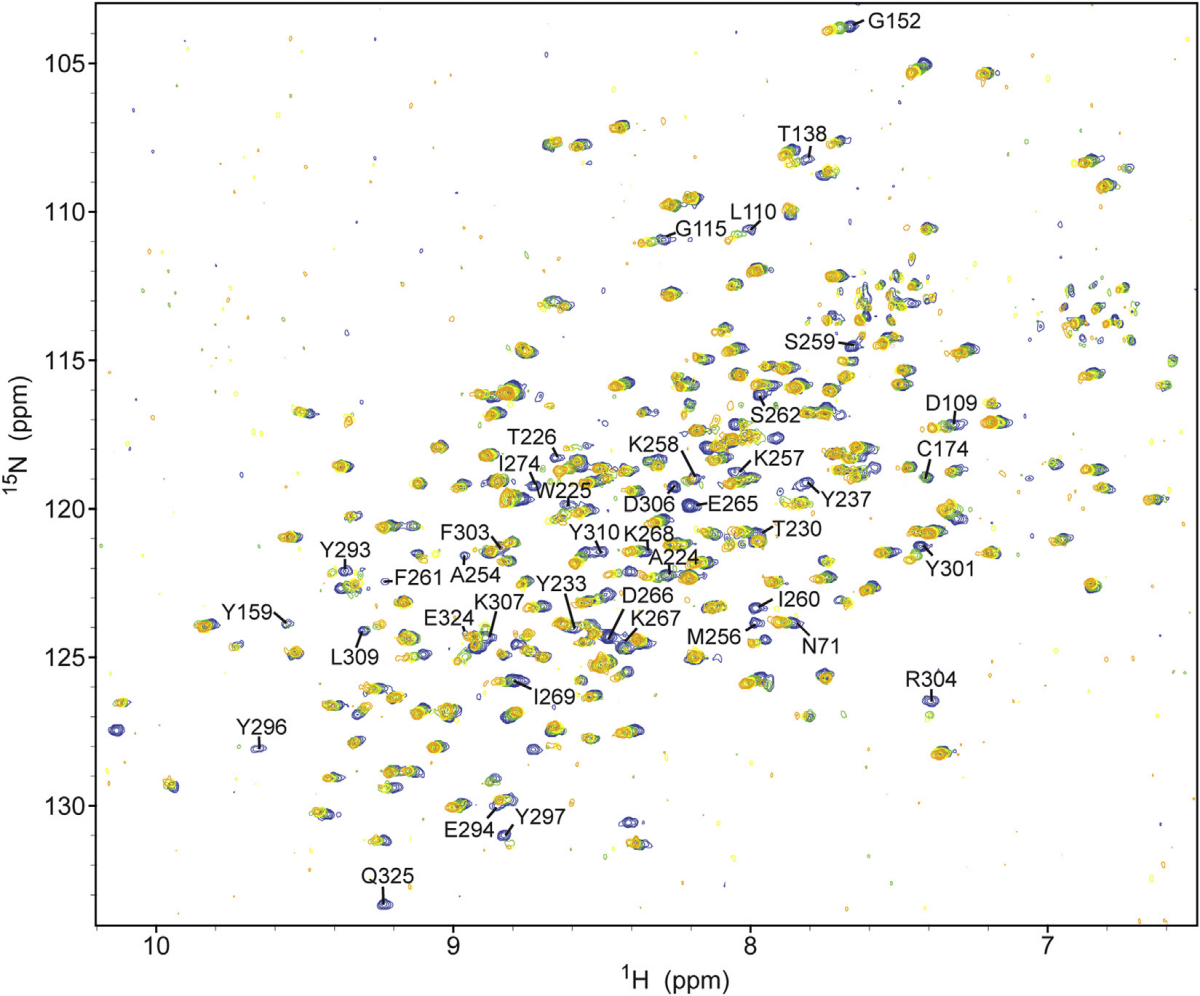
**Overlaid**^**15**^**N-BEST-TROSY spectra of**^**15**^**N-labeled Pgp2 titrated with muramidase-digested PG from *C. jejuni* Δ*pgp2*.** Spectra colored *blue*, *green*, *yellow*, and *orange* represent Pgp2 titrated with 0, 5, 10, and 20 μl of digested PG (60 μg/μl), respectively. Selected peaks from residues showing chemical shift perturbations or line broadening and signal loss over the course of the titration are labeled.

In general, the ^1^H^N^ and ^15^N chemical shifts of many amides in Pgp2 changed progressively with added ligand (Fig. 4). This corresponds to the fast exchange regime on the chemical shift timescale (k_ex_ >> Δω, where k_ex_ is the interconversion rate constant and Δω is the chemical shift difference between free and bound states) and is indicative of relatively weak binding (28). Such a response enabled their signals to be followed over the course of the titration, and their chemical shift perturbations (CSPs) to be calculated as the square root of the sum of the squared ^1^H^N^ and ^15^N chemical shift differences between the apo- *versus* ligand-bound protein at the titration end point (29). To define residues most perturbed by ligand binding, a CSP cut-off was determined for each given titration based on the average CSP value for all residues, combined with patterns of clustering when mapped to the structure. In some cases, increasing linewidths and decreasing intensities of amide signals also occurred upon ligand binding (Fig. S5). This typically corresponds to the intermediate exchange regime (k_ex_ ∼ Δω) and could arise from larger amide chemical shift changes upon binding, or perhaps sensitivity to exchange between multiple bound conformations. Although precluding the measurement of CSP values, the patterns of such spectral perturbations aided in the identification of ligand-binding sites.

**Figure 5:**
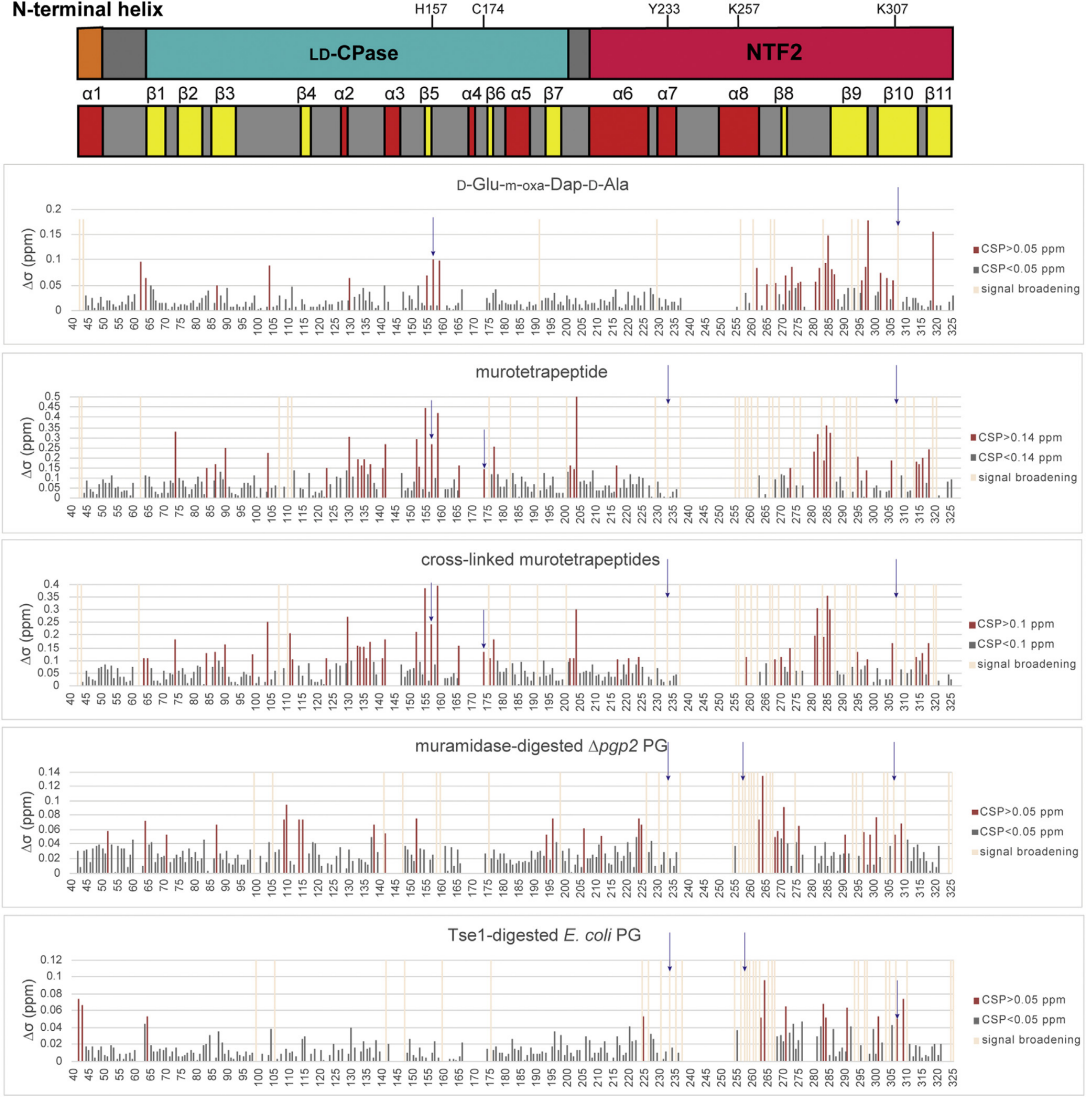
**Chemical shift perturbation (CSP) analysis of**^**15**^**N-labeled Pgp2 interacting with a panel of PG-derived ligands.** CSP values (Δδ) were calculated for each residue by $\Delta \text{δ}=\sqrt{{\left(\Delta {\text{δ}}_{\text{H}}\right)}^{2}+{\left(0.14*\Delta {\text{δ}}_{\text{N}}\right)}^{2}}$, where Δδ_H_ and Δδ_N_ denote the observed changes of the amide ^1^H^N^ and ^15^N chemical shifts in the absence *versus* presence of a PG ligand at the endpoint (highest) ligand:protein ratio for each given titration experiment (see Experimental procedures). Residues above and below the indicated CSP cut-off values are colored in *red* and *gray*, respectively. Residues that showed shift perturbations that could not be quantitated due to line broadening or severe intensity loss are colored in *light orange* (Fig. S5). Blank values correspond to prolines and residues with overlapping or unassigned signals. Residues that when mutated exhibited straight cell-shape phenotype (H157, C174, Y233, K257, K307) are marked with *arrow* in the bar graph.

**Figure 6:**
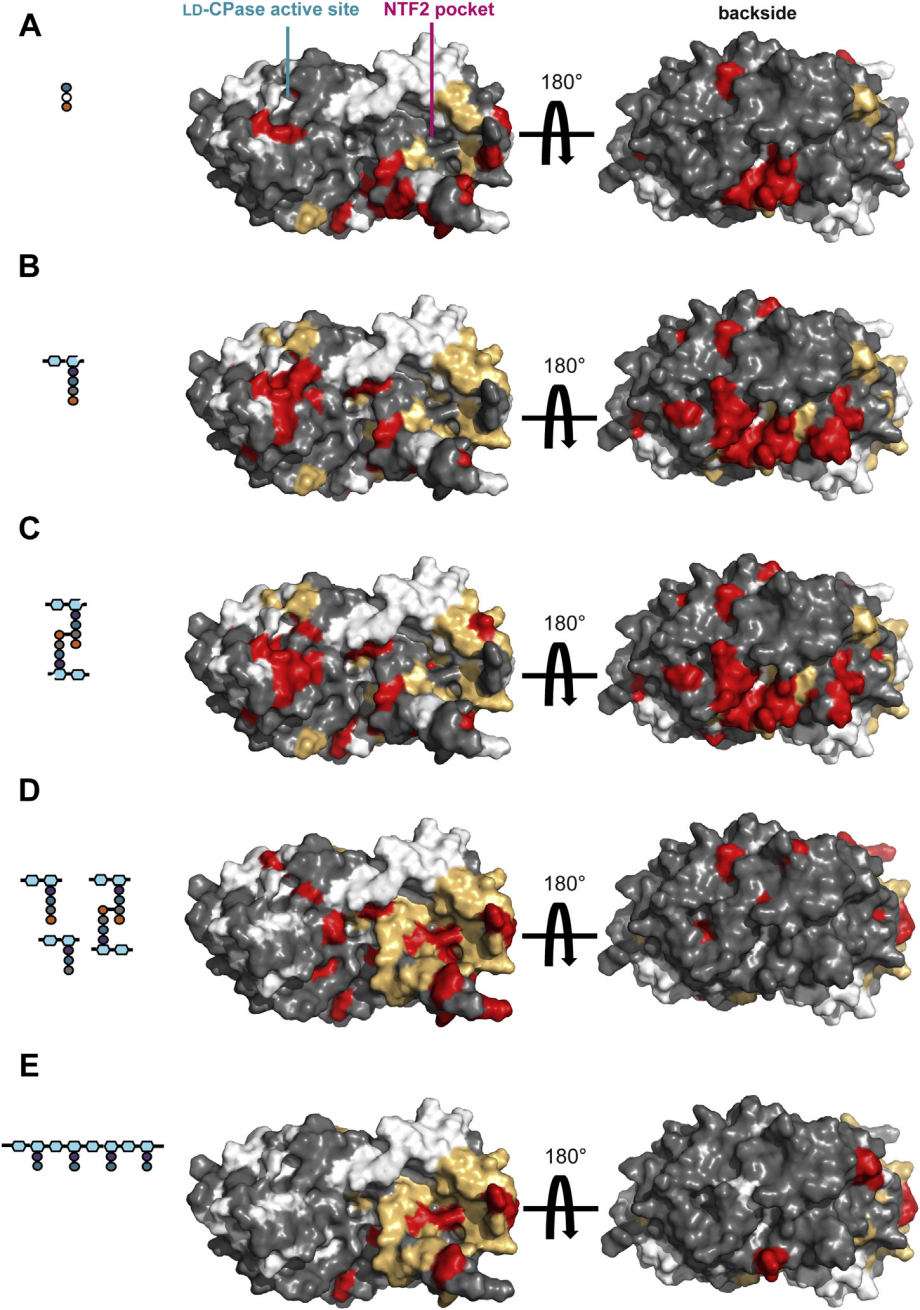
**Interaction surfaces of Pgp2 with PG ligands identified from NMR-monitored titrations.***A*, D-Glu-*m*-oxa-Dap-D-Ala. *B*, HPLC-purified murotetrapeptide. *C*, HPLC-purified cross-linked murotetrapeptides. *D*, muramidase-digested Δ*pgp2* PG. *E*, DL-endopeptidase (*P. aeruginosa* Tse1) digested *E. coli* PG. A representation of the ligand used in each experiment is shown as a diagram (*hexagons*: glycan backbone; *circles*: peptide residues; *open hexagons*: reduced MurNAc). Amide CSP values of ^15^N-labeled Pgp2 upon titration with the indicated PG ligand (Fig. 5) are mapped onto the protein surface (residues above the CSP cut-off in *red*; residues below the cut-off in *gray*; prolines and residues with unassigned signals in *white*). Also identified in orange are amides for which CSP values could not be determined due to severe signal line broadening (Fig. S5).

Titration with D-Glu-*m*-oxa-Dap-D-Ala identified three patches of amides in the protein with CSP values above the cut-off (Figs. 5 and 6*A*). These include a patch next to helix α2 and strand β5 of the active site in the LD-CPase domain (Y130, W155, H157, and Y159), consistent with slow hydrolysis of the synthetic substrate by Pgp2 (20). The second patch is located on the pocket of the NTF2 domain at helix α8 and strands β8–β10 (S262, E265, K268, F271, D273, N275, I276, Y296-T298, Q302, R304, and D306). A cluster of residues along helix α8 and strand β9 also showed signal broadening during the titrations (M256, I260, D266, K267, F292). This confirms the role of the NTF2 domain in PG binding. The third small patch is primarily from a loop with low sequence conservation at the inter-domain interface (N281, L282, and N284-M287) along the surface on the opposite side of both the LD-CPase domain active site and the NTF2 domain pocket.

Titrations using purified murotetrapeptide resulted in amides with line broadening or CSPs above the cut-off localized to three main patches (Figs. 5 and 6*B*). One patch, which includes helix α2, strand β5, and nearby loops (F123, Y130, F133–F135, T137, G152, G153, W155, H157, Y159, L166, C174, and L177), corresponds to a more extended interface near the Pgp2 active site than seen with the synthetic peptide. The second patch lies on the NTF2 pocket with residues within helices α7–α8 and strands β9–β11 showing extensive line broadening. The third backside patch is located at loops between the two domains (N281-L282 and N284-T286, E202-I204, D314-K316, K318) and is more extended than seen with the peptide titration. Titration of purified cross-linked murotetrapeptides identified the same three patches (Figs. 5 and 6*C*). No significant differences in the patterns of amide spectral perturbations were observed between monomeric murotetrapeptide and cross-linked murotetrapeptides. This suggests that, even when cross-linked, the murotetrapeptides (and the synthetic peptide) bind to distinct interaction surfaces on Pgp2.

A titration with muramidase-digested Δ*pgp2* PG identified a predominant patch within the NTF2 pocket that includes residues A224, W225, K268, I269, Y297, and K307, which are located along helix α6 and strands β8–β10 within the NTF2 pocket (Figs. 4, 5, and 6*D*). Considerable resonance broadening was also observed for the nearby residues, including Y233 and Y237 of helix α7, A254 and M256-S262 of helix α8, E265-K267, and residues of strands β9–β11. An analysis of peak intensity changes over the course of the titration revealed the largest reductions for amides on strands β8–β10 of the NTF2 domain (I269-F271, D273, F292, Y297, K299, Y301, and K307) (Fig. S5). Thus, under the conditions of this titration experiment, the digested PG mixture primarily bound to the NTF2 pocket.

Titration with Tse1-digested *E. coli* PG showed patterns of small CSPs for amides located at helices α6 and α8 (T220, W225, R263, and K264) and strands β8–β10 (F271, D273, I276, L282-N284, S291-F292, Y301, G305, K307, and L309) along the NTF2 domain pocket (Figs. 5 and 6*E*). In addition, resonance broadening was associated with residues on helices α6–α8 and strands β9–β11. Thus, Tse1-digested *E. coli* PG also bound to the pocket of the NTF2 domain, with little measurable association near the active site of the LD-CPase domain. This may reflect a relatively low endpoint concentration of the PG used for the titration and a decrease in tetrapeptides due to the cleavage mechanism of Tse1 (30). As perturbations of resonances of residues in the NTF2 pocket were observed by NMR when titrating with PG fragments, including residues that when mutated gave rise to a straight phenotype (Y233, K257, K307), binding of PG by the NTF2 domain may be a necessary for *C. jejuni* helical shape generation.

### NMR data-driven docking to identify binding modes between Pgp2 and PG

NMR-monitored titrations identified three major PG-binding sites: the catalytic cleft, the NTF2 pocket, and a backside patch. The titration with purified murotetrapeptide exhibited the largest continuous patch of perturbed residues in the catalytic cleft and the backside patch, and the muramidase-digested PG mixture displayed extensive perturbations in the NTF2 pocket. These two NMR titration data sets were therefore chosen for computational docking experiments using HADDOCK (31, 32) to construct a model of the Pgp2-PG complex. To overcome the inherent challenges of identifying bound conformations of a flexible ligand in molecular docking, an ensemble of murotetrapeptide conformers was derived by sampling molecular dynamic simulations using CNS. In addition, we used two Pgp2 crystal structures (WT and Pgp2^K307A^) as initial docking conformers.

**Figure 7:**
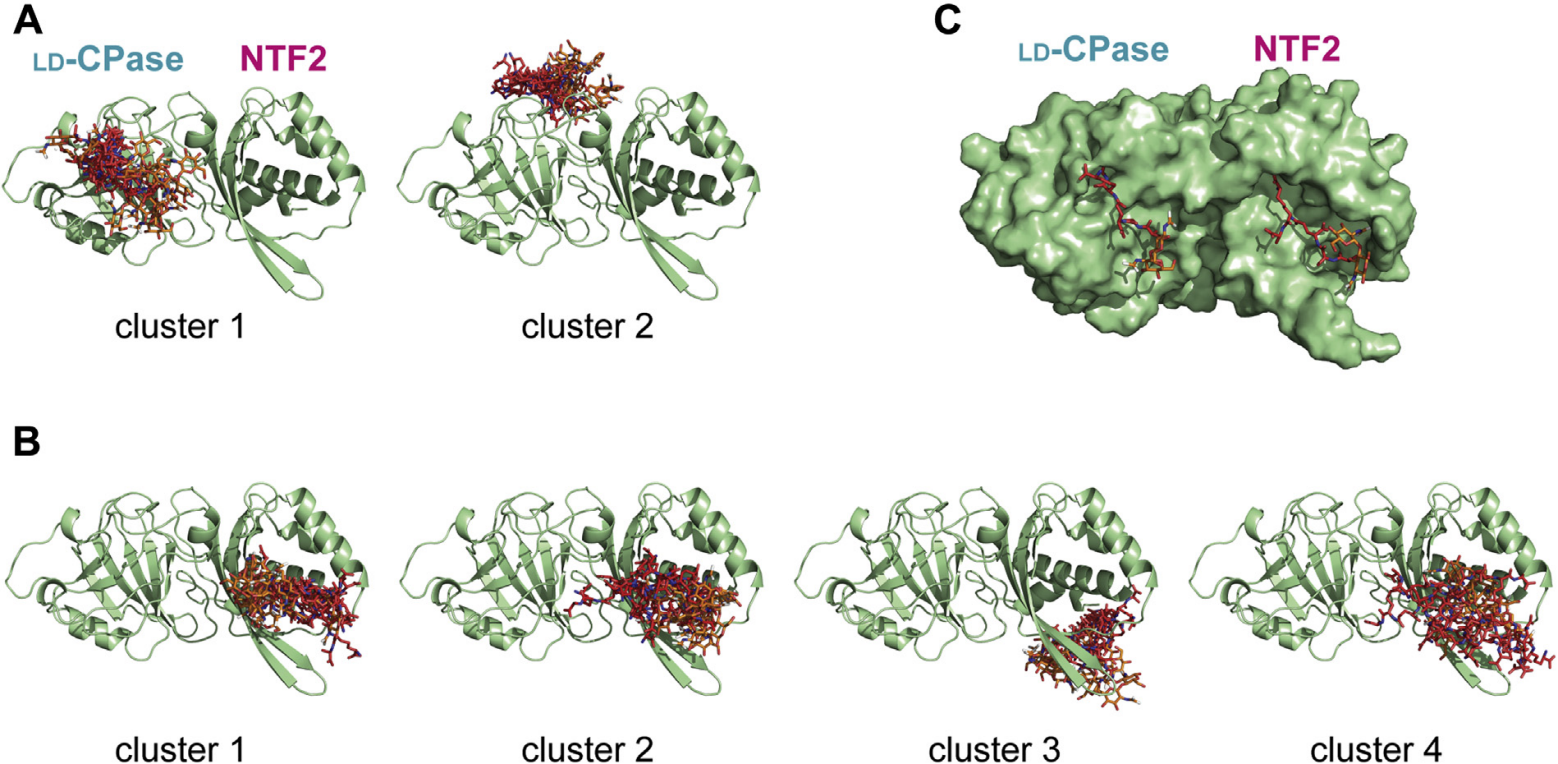
**HADDOCK models of the Pgp2-murotetrapeptide complexes driven by NMR spectral perturbations.** The top ten scoring docking solutions from each of the dominant clusters (with >10 solutions) in each docking experiment are displayed. Pgp2 is shown in *green*. The bound murotetrapeptide is shown in stick form, where the sugar and peptide moieties are colored *orange* and *red*, respectively. *A*, clusters identified from docking using CSP data from the murotetrapeptide titration experiment. *B*, clusters identified from docking using CSP data from the muramidase-digested PG titration experiment. *C*, superimposition of the best HADDOCK scoring models for the murotetrapeptides (*stick form*) bound to the LD-CPase and NTF2 domains.

For the docking using CSP data from the murotetrapeptide titration experiment, an unambiguous distance restraint of 2.0 Å between the nucleophile (Cys174) and the carbonyl carbon of *m*-DAP was added. The top 200 docking solutions were grouped into five clusters with a coverage rate of 92.5% (185/200) (Table S2). The largest cluster (88 solutions) had the best HADDOCK score with reasonable distances between nucleophilic C174 and the carbonyl carbon of *m*-DAP (Fig. 7*A*). Contacts within the complex were primarily between the active cleft and peptide moiety, with minor interactions between the protein and sugar moiety. Within this cluster, all disaccharides point away from the active cleft. The second major cluster (68 solutions) features contacts primarily between the murotetrapeptide and loops of the domain interface on the backside of Pgp2 (Fig. 7*A*). The solutions in this cluster are catalytically unfavorable because the nucleophilic attack distance for C174 is over 18 Å.

For docking using the CSP data from the titration with muramidase-digested PG, no unambiguous distance restraints were included. The top 200 solutions were grouped into five clusters with a coverage rate of 95.5% (191/200) (Table S2). The largest cluster (126 solutions) had the best HADDOCK score, featuring strands β8–β12 and helix α8 of the NTF2 domain pocket interacting with the peptide moiety (Fig. 7*B*). The contact regions within the second cluster (31 solutions) are similar to cluster 1 but position the backbone sugar in the reverse direction. The remaining clusters had poor scores with small buried surface areas.

**Figure 8:**
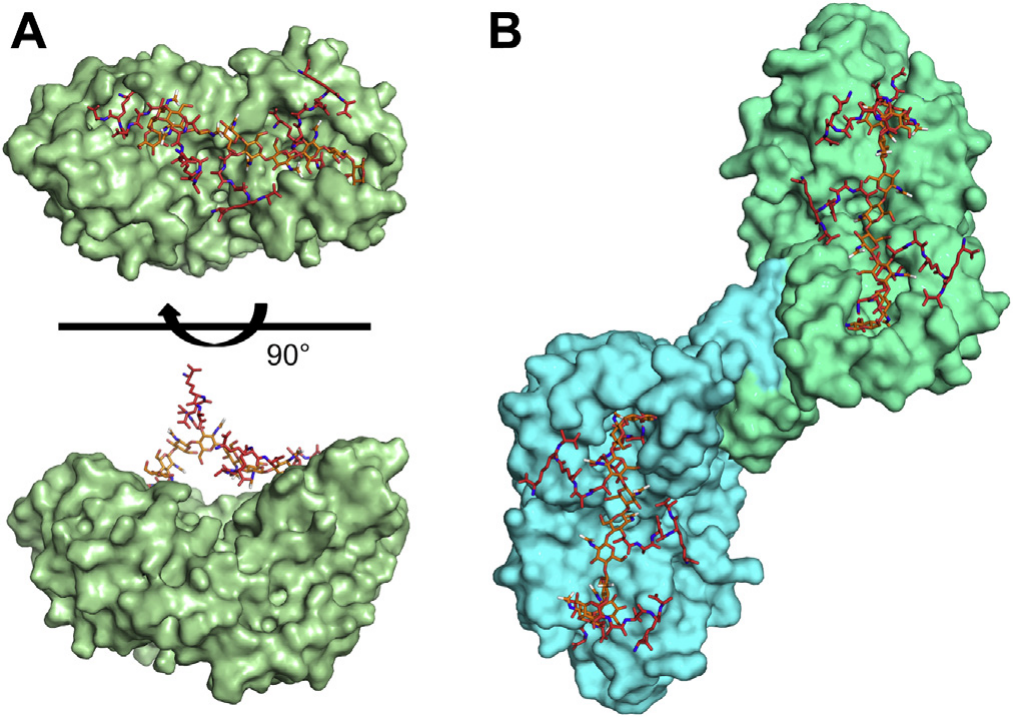
**The Pgp2-PG-binding model.***A*, A Pgp2-PG -binding model manually built by bridging the murotetrapeptides bound to the LD-CPase and NTF2-binding sites through a penta-disaccharide peptidoglycan polymer. Each MurNAc residue is attached to one tetrapeptide. PG is shown in *stick form*. The sugar is colored *orange* and tetrapeptide is colored *red*, respectively. Pgp2 is shown as a surface representation. *B*, a dimer Pgp2-PG-binding model made by superimposition of two copies of the Pgp2-PG model onto the dimeric Csd6 crystal structure (PDB ID: 4XZZ). The monomers are colored *cyan* and *green*, respectively.

Selecting the best solutions from the docking of a murotetrapeptide to the catalytic and NTF2 domains (Fig. 7*C*), we generated a model of Pgp2 bound to PG by manually building a bridging PG polymer between the two docked muropeptides, which are ∼40 Å apart (Fig. 8*A*). This PG strand runs the length of the Pgp2 molecule. Csd6 is a dimer formed by a small dimerization domain at the N terminus. Much of this domain is absent in the recombinant Pgp2 construct used in these biochemical studies. Based on homology to Csd6, full-length Pgp2 is expected to also form a dimer. To model PG binding to the Pgp2 dimer, two Pgp2-PG complexes were superimposed onto crystal structure of the Csd6 dimer (PDB ID: 4XZZ) (Fig. 8*B*). Remarkably, the two PG strands are on one face of the dimer and run antiparallel 32 Å apart, close to the interstand distance of model cross-linked PG (33).

## Discussion

Pgp2 is annotated to be within the YkuD protein family (PF03734) (34), which are LD-transpeptidases (LD-TPase) with a conserved catalytic triad of Cys, His, and the main-chain carbonyl of a third residue (Gly in Pgp2) (35). LD-TPases bind two muropeptide stems in the active cleft to catalyze the formation of (L) *m*-Dap^3^➝(D) *m*-Dap^3^ (3) cross-links (36). However, the catalytic cleft of Pgp2 can only accommodate a single muropeptide and a water molecule for peptide bond hydrolysis. Superimposition of the LD-CPase domain of Pgp2 and the LD-TPase domain of LdtMt5 from *M. tuberculosis* (PDB ID: 4Z7A) revealed that the Pgp2 loops composed of residues 102–115 and 138–151 block the entry of an acyl receptor, consistent with the absence of 3–3 cross-links in *C. jejuni* PG (6).

The catalytic domain of Pgp2 shares conserved features with Csd6, the LD-CPase from *H. pylori* (Fig. S3*E*). The arrangement of the catalytic triad (C174, H157, and G158) is conserved. Also conserved are residues (E107, Y130, and W155, Pgp2 numbering) interacting with two bound D-Ala molecules observed in the Csd6 active site (19). One D-Ala is proposed to mimic the binding of the D-Ala residue of the peptide stem substrate, whereas the second D-Ala is thought to bind in the *m*-DAP subsite. Despite these similarities, Pgp2 cleaved both monomeric and cross-linked tetrapeptides in a biochemical assay with recombinant enzyme containing the predicted dimerization domain (residues 19–325) and purified *C. jejuni* PG (7), whereas the equivalent Csd6 construct (residues 18–303) fully cleaved monomeric tetrapeptides with only trace digestion of cross-linked tetrapeptides in purified *H. pylori* PG (15). We assayed the same purified *H. pylori* Csd6 construct with *C. jejuni* PG and observed digestion of both monomeric and cross-linked tetrapeptides (Fig. S2*B*). Taken together, the difference in activity may thus be due to the differing PG architectures as opposed to enzyme substrate specificity differences.

Our pull-down data demonstrate that both the LD-CPase and NTF2 domains of Pgp2 are required for high-affinity binding to PG (Fig. S2*A*), a feature not previously observed for the LD-CPase enzyme family. Many cell wall enzymes contain a noncatalytic NTF2 domain in addition to their catalytic module. For example, an NTF2 domain is found in a subset of class B penicillin-binding proteins (16, 37), the DD-endopeptidase NlpD, and β-lactamase (17). These NTF2 domains may also be required for high-affinity PG binding, playing a role in the recognition of specific local PG structural features and guiding catalysis. Characterizing the structures and substrate preferences of the NTF2 domain from these enzymes will determine how this domain is adapted to diverse roles in PG metabolism.

We observed extensive NMR spectral perturbations clustered into three regions on the surface of Pgp2 upon titration with model PG ligands (*i.e.*, the CPase active site, the NTF2 pocket, and a third backside patch; Fig. 6, *A*–*C*). The conserved CPase active site and NTF2 domain pocket are ∼40 Å apart along the same side of the protein surface. The glycan backbone of PG was proposed to preferentially form a right-handed helix with a periodicity of 30–40 Å, or approximately three to four GlcNAc-MurNAc repeats with successive peptide stems projecting outward from the glycan strand screw axis (38, 39). As the top solutions from NMR-data driven docking at both domains position two bound murotetrapeptides oriented such that they can originate from a single right-handed PG strand (Fig. 7*C*), we propose this as the most parsimonious Pgp2-PG binding model, an interaction that simultaneously involves both the catalytic and NTF2 cavities (Fig. 8*A*).

The backside patch features low sequence conservation and was prominent during titrations with the synthetic peptide analog and purified monomeric and cross-linked murotetrapeptides (Fig. 6, *A*–*C*). Situated between the two domains, the region may be involved in allosteric coupling of the two frontside binding sites. Neither the third patch nor the active site exhibited strong relative perturbations when titrated with enzymatically digested PG (Fig. 6, *D* and *E*). This may reflect low relative concentrations of each muropeptide in the PG mixture and complex binding dynamics arising from multiple binding sites.

Pgp2 likely forms a higher oligomerization state within the bacterial cell. The Pgp2 homolog Csd6 dimerizes through three N-terminal helices from each monomer, building a six-helix bundle (19). The hydrophobic dimer interface of Csd6 is composed of the sequence **IM**R**LY**X_3_G**L**E**MV**. The N-terminal residues, including the hydrophobic dimer interface (**LV**R**IY**X_3_G**L**D**AV**), are conserved in Pgp2 but missing in the truncated recombinant construct. We used the crystal structure of the Csd6 dimer (PDB ID: 4XZZ) and our PG docking data to predict the interaction of a Pgp2 dimer with PG (Fig. 8*B*). In this model, the PG interaction interface of both Pgp2 monomers is on the same face of the dimer. The Pgp2 dimer can interact with two PG strands ∼32 Å apart, allowing for recognition of an ordered PG tertiary structure that may direct patterned digestion of the PG to form helical shape.

Some Pgp2 and PG structural features were not included in the generation of the proposed model. Firstly, the docked murotetrapeptides did not include acetylation at O6 of MurNAc. Secondly, strands β9–β10, which form the lip of the NTF2 pocket, are flexible (Fig. S3, *A–D*), and this flexibility may alter binding specificity. Lastly, a more complex PG-binding model is possible in which the two muropeptides bound to the active site and NTF2 domains originate from separate glycan strands ∼40 Å apart. In this alternate model the Pgp2 dimer could interact with up to four distinct PG strands. Solid-state NMR can be used to calculate the ^13^C-^15^ N internuclear distance of D-[1-^13^C]Ala and L-[^15^N]Ala -labeled PG peptide stems (40) and may be able to resolve these possibilities. Attempts to cocrystallize Pgp2 with synthesized peptide D-Glu-*m*-oxa-Dap-D-Ala and purified murotetrapeptide were ultimately unsuccessful.

Bacterial cell shape requires the spatial coordination of PG insertion (41). A localized “shapesome” complex, coordinated across the cytoplasm, inner membrane, and periplasm, is proposed to contribute to helical shape generation in *H. pylori via* asymmetrical cell wall synthesis (42, 43, 44). However, no interactions were identified between Pgp2 and PG synthesis machinery. Biophysical modeling suggests that spatially targeted PG growth (45, 46, 47) or changes to the degree of cross-linking along a helical axis can lead to a helical cell shape (48, 49). Neither overall growth rates (7) nor cell length (Fig. 2*B*) was affected upon the deletion of Pgp2. However, Pgp2 may modulate spatial PG insertion or cross-linking. The percentage of cross-links are similar between the wild-type (47.9%) and Δ*pgp2* (47.6%) strains (7), suggesting that the overall proportion of cross-links is not sufficient to determine helical shape. Instead, the deletion of Pgp2 increases the ratio of tetra-tetra to tetra-tri cross-links due to the absence of tripeptides (7). In *H. pylori*, the overall proportion of cross-links is also unaffected upon the deletion or overexpression of Csd6, but the ratio of tetra-tetra to tetra-tri cross-linked peptide stems varies (15). Both deletion and overexpression of Csd6 give a straight cell shape, suggesting that helical shape requires a proper balance between these two types of cross-links.

We show that Pgp2 has higher enzymatic activity on monomeric as compared with cross-linked peptides (Fig. 3), consistent with previous experiments using synthetic model peptides and purified PG (7, 20). By mutating conserved NTF2 domain pocket residues, we demonstrate that this domain impacts enzyme activity and is required for the preference for monomeric substrates in *C. jejuni*. Point mutations in the NTF2 domain resulted in a 75–86% reduction in the proportion of monomeric tripeptide products in *C. jejuni* PG as compared with wild-type. The same mutations reduced cross-linked tetratripeptides by 5–59% in the same samples. Clearly, muropeptide distribution is insufficient to determine shape phenotype. The NTF2 domain mutants (K257A, K307A, E324Q) have a similar loss in monomeric and cross-linked tripeptides but differ in shape phenotype ranging from curved to straight rods (Fig. 2, *A* and *B*). Nonetheless, these mutants may differ in function, such as modified localization or targeting of PG substructures, which would lead to the shape phenotypes.

Based on the evidence that NTF2 domain binds to PG and regulates Pgp2 activity, we propose that preferential trimming of monomeric tetrapeptides by Pgp2 is localized to one of the helical axes. Since tripeptides produced by Pgp2 can be further digested to release *m*-DAP by the DL-carboxypeptidase Pgp1 (7), they are no longer able to form cross-links. Thus, Pgp2 activity along a helical axis may allow for local relaxation of the PG, leading to cell twist. Future examination of Pgp2 localization and involvement of the NTF2 domain will test this model.

The requirement of Pgp2 for helical shape may serve as a basis for antimicrobial development as *C. jejuni* colonization within the host could be reduced. This strategy was successfully employed to target Pgp1 and the *H. pylori* homolog Csd4 (50). Incubation of bacteria with a small-molecule inhibitor of the carboxypeptidase domain results in a morphological shift from helical to straight rod cells. Unlike the LD-CPase domain, which is found in both helical and nonhelical-shaped cells, the NTF2 domain appears to be restricted to helical-shaped cells, suggesting that the NTF2 domain may be a more selective target.

In summary, we show that helical shape in *C. jejuni* depends on both the LD-CPase and NTF2 domains of Pgp2. Our proposed Pgp2-PG model highlights the importance of PG binding by the NTF2 domain that may guide Pgp2 activity through recognition of PG architecture.

## Experimental procedures

### Crystal structure determination

The Pgp2 and Pgp2^K307A^ structures were determined by molecular replacement. The methods used for recombinant protein expression, crystallization, and structure determination are described in Text S1 (supporting information).

### Complementation with wild-type and site-directed mutagenesis variants Pgp2 in a Δ*pgp2* strain

Complementation of Δ*pgp2* was achieved using a *pgp2* complementation plasmid (7). The *pgp2* gene and promoter were amplified from *C. jejuni* 81–176 genomic DNA and cloned into the pRRC vector (51). The inserted *pgp2* gene included 196 bp of the native promoter region and 190 bp of the downstream sequence. The complementation plasmid was integrated into an available strain 81–176 Δ*pgp2* mutant (7) by natural transformation. The transformed *C. jejuni* cells were grown at 38 °C under microaerophilic conditions (12% CO_2_, 6% O_2_, in N_2_) in a trigas incubator. Colonies were selected on MH-TV plates with chloramphenicol. Recombination into the chromosome was verified by PCR analysis using primers Spe1, 198R, 554F, and cat-2. Construction of variant Pgp2 mutant strains was achieved by site-directed mutagenesis of the wild-type complementation plasmid. Immunoblotting to determine Pgp2 expression levels is described in Text S1. The primers used and bacterial strains used in this study are listed in Table S3.

### Microscopy and celltool shape analysis

Overnight broth cultures of *C. jejuni* were standardized to OD_600_ 0.05 in fresh MH-TV media and grown for 4 h at 38 °C under microaerophilic conditions (Oxoid CampyGen) to log phase (OD_600_ 0.1–0.3). Cells were mounted on a 1% agarose gel pad on a glass slide and imaged with a Nikon Eclipse TE2000-U microscope equipped with 100× oil-immersion objective and a Hamamatsu C4742-95 digital camera. Cell images were transformed into binary mode with GIMP software and analyzed with the Celltool software package (24). PCA analysis on wild-type cell contours of ∼400 cells from each strain generated a shape mode that described cell curvature from straight to highly curved morphology. To compare the curvature distributions of each strain to the distribution of wild-type cells, all cells were aligned with the averaged morphology of wild-type cell by iterative translation and rotation.

### Preparation of peptidoglycan and muropeptides

*C. jejuni* was cultured on 100 MH-T plates for 18 h at 38 °C, and at late log phase cells were collected in cold MH-TV broth (1 ml/plate) by scraping. Culture medium was removed by centrifugation at 5000 rpm at 4 °C for 10 min and the cell pellet was resuspended in 80 ml cold PBS buffer. Cells were lysed by mixing with an equal volume of boiling 6% SDS for 4 h and centrifuged at room temperature to remove intact cells. The PG was pelleted from the cell lysate by ultracentrifugation at 45,000 rpm at 22 °C for 3 h and washed with water. To remove glycogen and lipoproteins, the PG was resuspended in buffer (10 mM Tris pH 7.0 and 10 mM NaCl) and digested sequentially with alpha-amylase (200 μg/ml) and Pronases (200 μg/ml) at 37 °C overnight. The PG sample was boiled in 6% SDS for 10 min followed by centrifugation (10,000 rpm, room temperature, 10 min). SDS was removed by three washes with water and ultracentrifugation (45,000 rpm at 22 °C for 3 h). PG samples were lyophilized in water and stored at –20 °C. To prepare muropeptides, 0.2 mg PG was digested with 50 U mutanolysin (Sigma) in 50 mM Tris pH 7.0 and 150 mM NaCl at 37 °C for 24 h. Mutanolysin was removed by boiling for 10 min and centrifugation. Muropeptides prepared for HPLC analysis were reduced with 100 mM sodium borohydride pH 9.0 at room temperature for 30 min, titrated to pH ∼4 with phosphoric acid, and passed through a 0.22 um filter.

### HPLC muropeptide analysis

Muropeptide separation by HPLC was performed with an Xterra MS C18 column (Waters) and UV detection at 210 nm. Elution relied on a gradient from 100% buffer A (sodium phosphate buffer 50 mM pH 4.3) to 100% buffer B (sodium phosphate buffer 50 mM pH 4.9 and methanol 15% (v/v)) over 100 min.

### NMR titration experiments

NMR titration experiments were performed with ^15^N-labeled Pgp2. The assignment of Pgp2 amide resonances was achieved with ^2^H-^13^ C-^15^N Pgp2 as described in the supplemental Text S1. PG ligand titration studies with ^15^N-labeled Pgp2 were monitored by ^15^N-BEST-TROSY-HSQC at 25 °C for different ligand-to-protein ratios. PG ligand was dialyzed against water using a float dialysis membrane device with a 100–500 Da cut-off (Spectrum Laboratories, Inc) and lyophilized before preparation of a concentrated stock solution in NMR buffer (52). A 160 μM sample of ^15^N-labeled Pgp2 was titrated with 3.4 and 6.8 μl of D-Glu-*m*-oxa-Dap-D-Ala (4 μg/μl; MW 434.2 g/mol), giving a final peptide:protein molar ratio of 2:1. A 160 μM sample of ^15^N-labeled Pgp2 was titrated with 5, 10, and 20 μl of purified murotetrapeptide (50 μg/μl; MW 941.1 g/mol) to a final peptide:protein molar ratio of 33:1. A 160 μM sample of ^15^N-labeled Pgp2 was titrated with 5, 10, and 20 μl of purified cross-linked murotetrapeptides (50 μg/μl; MW 1864.8 g/mol) to a final peptide:protein molar ratio of 16:1. A 160 μM sample of ^15^N-labeled Pgp2 was titrated with 5, 10, and 20 μl of muramidase-digested Δ*pgp2* PG solution (60 μg/μl) to a final concentration of 5.5 μg/μl. A 110 μM sample of ^15^N-labeled Pgp2 was titrated with 60 μl of Tse1-digested *E. coli* PG (∼30 μg/μl) to a final concentration of ∼4 μg/μl. Spectra were overlaid in NMRFAM-SPARKY, and CSP (Δδ) values were calculated as $\Delta \text{δ}=\sqrt{{\left(\Delta {\text{δ}}_{\text{H}}\right)}^{2}+{\left(0.14*\Delta {\text{δ}}_{\text{N}}\right)}^{2}}$, where Δδ_H_ and Δδ_N_ denote the observed changes of the amide ^1^H^N^ and ^15^N chemical shifts in the absence *versus* presence of PG ligand at the final highest concentration used for a titration series.

### Data-driven docking

Models of Pgp2 in complex with murotetrapeptides were produced in HADDOCK 2.2 (31). Two starting conformers for Pgp2, based on the wild-type and Pgp2^K307A^ crystal structures, were generated as they differed in the NTF2 loop region that overhangs the pocket entrance. Unmodeled side chains were manually rebuilt and residue 307 was restored to Lys in Pgp2^K307A^ using Coot (53) prior to docking. The sugar moiety coordinates and topology files of GlcNAc-GlcNAc with a β-1,4 glycosidic bond with a phi/psi angles of 69°/12° (38) were produced with the GlyC_a_NS server (54). Tetrapeptide coordinates and topology files were generated with the PRODRG server (55) using a tetrapeptide model extracted from PDB entry 2MTZ. An ensemble of 20 murotetrapeptide conformers were generated by simulated annealing and energy minimization (56).

Ambiguous Interaction Restraints (AIRs) are defined as either active residues involved in binding or passive residues potentially involved (Table S2). The active residues of Pgp2 were defined as solvent accessible residues with a CSP above a cut-off in the murotetrapeptide titration experiment and from the list of functional residues identified by mutagenesis. The passive residues of Pgp2 were defined as the proximal residues within 5 Å of the active residues. The active residues of the murotetrapeptide were the peptide moiety and the passive residues were the sugars. For the docking experiment using data from a titration with purified murotetrapeptide, an unambiguous distance restraint (2.0 Å) was defined between the sulfur of the nucleophile Cys174 and the carbonyl carbon of *m*-DAP. A sample of 10,000 docking solutions were generated at the rigid body stage. The top 400 complexes based on HADDOCK score were used subjected to simulated annealing and the resulting top 200 complexes were further refined with waters. The docking solutions formed five clusters using a fraction of common contacts (FCC) cut-off of 0.45. In a second docking experiment, the active residues of Pgp2 were defined from residues with CSP above the cut-off in the muramidase digested Δ*pgp2* PG titration experiment. Both the peptide and sugar moieties of the murotetrapeptide were defined as passive residues. The final 200 solutions were grouped into five clusters using the FCC cut-off of 0.4.

## Data availability

The atomic coordinates and structure factors of Pgp2 and Pgp2^K307A^ have been deposited in the Protein Data Bank under ID codes 6XJ6 and 6XJ7. Pgp2 chemical shift assignments have been deposited to the Biological Magnetic Resonance Data Bank under ID 50689.

## Supporting information

This article contains supporting information (57, 58, 59, 60, 61, 62, 63, 64, 65).

## Conflict of interest

The authors declare that they have no conflicts of interest with the contents of this article.
