# Supplementary material for: Peptidoglycan binding by a pocket on the accessory NTF2-domain of Pgp2 directs helical cell shape of Campylobacter jejuni
Source: J Biol Chem. 2021 Mar 10;296:100528. doi: 10.1016/j.jbc.2021.100528 (PMC8038945; doi:10.1016/j.jbc.2021.100528)
Supplement: Text S1, Tables S1 to S3 and Figures S1 to S5 [file mmc1.pdf]

Peptidoglycan binding by a pocket on the accessory NTF2-domain of Pgp2 directs helical cell shape of *Campylobacter jejuni*

Chang Sheng-Huei Lin[1], Anson C. K. Chan[1], Jenny Vermeulen[1], Jacob Brockerman[2], Arvind S. Soni[3], Martin E. Tanner[3], Erin C. Gaynor[1], Lawrence P. McIntosh[2,3], Jean-Pierre Simorre[4], Michael E. P. Murphy[1*]

[1]Department of Microbiology and Immunology, University of British Columbia, Vancouver, British Columbia V6T 1Z3, Canada

[2]Department of Biochemistry and Molecular Biology, University of British Columbia, Vancouver, British Columbia V6T 1Z3, Canada

[3]Department of Chemistry, University of British Columbia, Vancouver, British Columbia V6T 1Z1, Canada

[4] University of Grenoble Alpes, CNRS, CEA, IBS, 38000 Grenoble, France

* To whom correspondence should be addressed: Dept. of Microbiology and Immunology, 2350 Health Sciences Mall, University of British Columbia, Vancouver, BC V6T 1Z3, Canada., Tel.: 604-822-8022; Fax: 604-822-6041; E-mail: michael.murphy@ubc.ca

Supporting information

Text S1. Supplemental methods

Table S1. Data collection and refinement statistics for Pgp2.

Table S2. HADDOCK calculation statistics.

Table S3 Strains and primers used in this study.

Figure S1. Western blots of whole cell lysates of *C. jejuni* strains probed with anti-Pgp2 polyclonal antibodies.

Figure S2. Binding of Pgp2 mutants to Δ*pgp2* PG and digestion by Csd6.

Figure S3. Superimpositions of Pgp2, Pgp2^{K307A} and Csd6 structures.

Figure S4. HSQC spectra of Pgp2 assignment of backbone amide ^{15}N and $^{1}H^{N}$ cross resonances.

Figure S5. Relative intensity analysis of ^{15}N-labeled Pgp2 interacting with a panel of PG derived ligands.

Text S1. Supplemental methods

Expression and purification of *C. jejuni* Pgp2. A region of the *pgp2* (*cjj81176_0915)* coding sequence corresponding to the product without the N-terminal signal peptide (residue 1-18) and the following 24 residues was cloned into a pET15 vector using *Nde*I and *Xho*I restriction sites, producing pET15b-Pgp2$^{43-325}$. The resulting recombinant Pgp2 protein includes an N-terminal His$_6$-tag followed by a thrombin cleavage site and residues 43-325. Unlabeled or labeled proteins were expressed in *Escherichia coli* BL21 (λDE3). Unlabeled recombinant Pgp2 was expressed in Luria-Bertani (LB) broth and purified as previously described (1).

^{15}N-labeled Pgp2 was produced in modified M9 minimal media (6 g/L Na_2HPO_4, 3 g/L KH_2PO_4, 0.5 g/L NaCl, 1 g/L $^{15}NH_4Cl$, 10 g/L D-glucose, 1 mM $MgSO_4$, 10 mg/L vitamin B1, 10 µM $FeCl_3$, 0.1 mM $CaCl_2$, 100 µg/mL ampicillin). Briefly, overnight LB broth cultures were collected by centrifugation and resuspended in M9 media. Cell suspensions were standardized to OD_{600} 0.05 in M9 media and grown at 37 °C to achieve log phase (OD_{600} ~0.7-1.0). Protein expression was induced for 16 hours at 22 °C with 0.3 mM IPTG. The purification protocol was adapted from that for unlabeled Pgp2. The final buffer contained 50 mM Tris-HCl, pH 6.5, 150 mM NaCl and 1 mM dithiothreitol (DTT). This was the buffer for all NMR experiments in this study.

Pgp2 labeled with ^{2}H, ^{13}C, and ^{15}N was expressed in M9 minimal media with deuterium oxide (D_2O) and D-glucose-$^{13}C_6$-d_7 (3 g/L). Protonated reagents were dissolved in D_2O and lyophilized prior to use. To optimize expression, cells were first adapted to deuterium M9 media (2), inoculated in M9/D_2O media at OD_{600} 0.05, and cultured at 37 °C to OD_{600} 1.0. Protein expression was induced with 1 mM IPTG for 16 hours at 25 °C. Cells were lysed in denaturing buffer (4 M guanidinium HCl, 20 mM Na_2HPO_4, pH 7.0, 0.5 M NaCl, 2 mM Tris(2-carboxyethyl)phosphine (TCEP), and 5% glycerol) supplemented with 1 mM phenylmethylsulfonyl fluoride (PMSF) and DNase using a homogenizer. The denatured protein underwent uniform amide deuterium to protium exchange, as required for ^{1}H-detected NMR measurements. The cleared supernatant was loaded onto a HisTrap HP column (GE Healthcare). After binding, the column was washed with denaturing buffer followed by on-column refolding with gradually decreasing concentrations of guanidinium HCl to 0 M. Folded protein was eluted with elution buffer (20 mM Na_2HPO_4, pH 7.0, 0.5 M NaCl, 0.5 M imidazole, 2 mM TCEP, and 5 % glycerol). The His$_6$-tag was cleaved by thrombin (100:1 w/w Pgp2: thrombin ratio) during dialysis (50 mM Tris pH 7.0, 150 mM NaCl and 1 mM DTT). Thrombin was removed with p-aminobenzamidine agarose beads (5 mg thrombin: 1 mL beads).

Uncleaved proteins and cleaved His_6-tag were removed using a HisTrap HP column. His_6-tag free Pgp2 was polished using a Superdex 200 16/60 column (GE Healthcare) in NMR experiment buffer. The sample was immediately concentrated to ~ 350 uM and was used for NMR data collection.

The $Pgp2^{K307A}$ expression plasmid was made by site-directed mutagenesis using pET15b-$Pgp2^{43-325}$ as the template. $Pgp2^{K307A}$ protein was purified as described for recombinant Pgp2.

Crystallization and structure determination. Unlabeled recombinant Pgp2 (10 mg/mL) was prepared in a buffer of 50 mM Tris pH 7.0, 150 mM NaCl and 1 mM DTT. Pgp2 crystals grew at room temperature using the hanging drop vapor diffusion method. The reservoir contained 200 mM tris-methylamine N-oxide dehydrate, 100 mM Tris pH 8.5 and 20 % (w/v) PEG monomethyl ether 2000. Rectangular shaped crystals grew to size of 0.2-0.4 µm within a week. Reservoir solution supplemented with 25 % glycerol was used as a cryoprotectant. X-ray diffraction data was collected at the Stanford Synchrotron Radiation Light source at beamline 9-2. Each data set was collected from a single crystal at 100 K using a wavelength of 0.98 Å. Diffraction images were processed using HKL2000 (3). The Pgp2 structure was phased by molecular replacement method using Csd6 (PDB ID: 4XZZ) as the search model with Phenix.phaser (4). The structure was refined with Phenix.refine and manual model building was done with Coot (5). The final model includes residues Ser-His-Met from the expression vector and the residues of the Pgp2 construct (residues 43 to 325). Due to weak electron density, the side chains of residues R121, K268, Y296, Y297, Q302 and F303 were not included in the model. $Pgp2^{K307A}$ (14 mg/mL) was prepared in 50 mM Tris pH 7.0, 500 mM NaCl and 10 % glycerol. The reservoir contained 0.17 M ammonium acetate, 0.085 M sodium acetate pH 4.6, 25.5 % (w/v) PEG 4000 and 15 % (v/v) glycerol. $Pgp2^{K307A}$ crystals grew at 4 °C by hanging drop vapor diffusion and were cryoprotected with 20 % glycerol. X-ray diffraction data were collected from a single crystal at 100 K using a wavelength of 0.98 Å at the Canadian Light Source on beamline 08B1-1. Diffraction images were processed using XDS (6) and CCP4 packages (7), phased using the Pgp2 structure and refined as above. Data collection and refinement statistics are summarized in Table S1. The coordinates are deposited as entries 6XJ6 and 6XJ7 in the Protein Data Bank.

NMR spectral assignments. NMR spectra for resonance assignments of Pgp2 were recorded at 25 °C using an ~ 350 µM uniformly labeled ^2H-^{13}C-^{15}N Pgp2 sample with 5 % D_2O included for locking. Spectra were acquired on Bruker Avance III 500, 600, and 850 MHz spectrometers equipped with xyz-gradient TCI cryoprobes. Collected spectra include a ^{15}N-BEST-TROSY-HSQC, a BEST-TROSY-HNCACB/BEST-TROSY-HNCOCACB pair, and a BEST-TROSY-HNCACO/BEST-TROSY-HNCO pair (8). Data were processed with NMRpipe (9) and analyzed in NMRFAM-SPARKY (10) and Topspin. Spectra were automatically interpreted using PINE (11) and verified manually to assign main chain ^1H, ^{13}C, and ^{15}N signals.

Immunoblotting. *C. jejuni* whole cell lysates of each strain were prepared from log phase cultures (OD_{600} 0.1–0.3) that were centrifuged and frozen. The cell pellet was lysed in 100 µl of 0.1 % Triton X-100, 50 mM Tris pH 8.0, 10 % glycerol and 150 mM NaCl at room temperature for 30 min. Total protein from each lysate was by normalized to OD_{595} 0.44/µl using Bradford reagent (Bio-Rad). Sample were separated on a 12 % SDS-PAGE and transferred onto a nitrocellulose membrane. Membranes were blocked by TBS-T buffer plus 5 % skim milk (TBS-T; 20 mM Tris pH 7.5, 150 mM NaCl and 0.5 % Tween 20) at 4 °C for overnight. The membrane was incubated with 1:3500 dilution of an anti-Pgp2 antibody, produced from a rabbit immunized with recombinant Pgp2, at 4 °C overnight followed by three washes with TBS-T buffer. Secondary antibody Alexa Fluor 680 (Invitrogen) was added at 1:2000 dilution and incubated for 1 hour at room temperature. The membrane was washed three times with TBS-T buffer before imaged at 700 nm by Odyssey Classic Imaging System (LI-COR).

Table S1. Data collection and refinement statistics for Pgp2

	$Pgp2^{43\text{-}325}$	$Pgp2^{K307A}$
**Data collection**		
Space group	$P2_12_12_1$	$P22_12_1$
Cell dimensions		
a, *b*, *c* (Å)	47.5, 71.9, 91.7	64.9, 89.8, 106.6
α, β, γ (°)	90, 90, 90	90, 90, 90
Total reflections	320747	396576
No. of unique reflections	51069	53923
Resolution (Å)[a]	50.00-1.50 (1.53-1.50)	50.00-1.85 (1.89-1.85)
R_{merge}[a]	0.037 (0.581)	0.102 (0.868)
$I/\sigma I$ [a]	33.5 (2.0)	13.1 (2.3)
CC (1/2)	0.978 (0.885)	0.998 (0.829)
Completeness (%)[a]	99.5 (99.5)	100 (100)
Redundancy	6.3 (6.1)	7.4 (7.4)
**Refinement**		
Resolution (Å)	32.97-1.50	47.18-1.85
R_{work}/R_{free}	0.158/0.192	0.164/0.203
Ramachandran		
Favored (%)	98.2	99.1
Allowed (%)	1.8	0.9
Outliers (%)	0	0
Average B factors (Å^2)		
Protein	30.8	31.0
Water	38.5	35.6
RMSDs from ideal values		
Bond lengths (Å)	0.004	0.010
Bond angles (°)	0.709	0.964
PDB ID	6XJ6	6XJ7

[a]Value for the highest resolution shell is shown in parenthesis.

Table S2. HADDOCK calculation statistics.

Docking experiment	Murotetrapeptide		Muramidase-digested PG			
**AIR** [a]						
Pgp2 active residues [b]	74,87,111,266,273,282,284,286		64,87,110,138,152,225,262,264,268,293,297,299,301,304,306,324			
Pgp2 passive residues [b]	55,62,63,73,83,86,88,89,95,105,106,107,108,121,122,124,127,128,129,132,138,145,147,150,153,161,164,165,168,170,172,178,183,201,202,203,205,245,264,265,268,272,283,285,293,296,304,325		44,45,48,49,50,53,54,55,57,62,63,73,74,75,83,84,86,88,89,105,106,107,108,111,118,120,136,145,147,150,153,168,169,170,172,190,192,193,201,202,203,204,205,207,208,209,210,223,227,229,231,232,235,239,240,241,243,244,245,248,250,252,255,256,258,259,260,263,265,266,270,272,273,281,282,295,296,298,300,302,303,325			
murotetrapeptide active residues [b]	3		1, 2, 3			
murotetrapeptide passive residues [b]	1,2					
**Cluster**[c]						
Number	1	2	1	2	3	4
HADDOCK score	-52 ± 11	-35 ± 8	-81 ± 8	-54 ± 12	-59 ± 13	-30 ± 16
Average RMSD (Å) between structures	1.0 ± 0.5	0.8 ± 0.3	1.3 ± 0.5	0.8 ± 0.4	0.9 ± 0.4	0.99 ± 0.5
No. of structures	88	68	126	31	16	11
Buried surface area ($Å^2$)	810 ± 153	835 ± 118	1131 ± 116	1034 ± 138	833 ± 64	859 ± 143
$E_{intermolecular}$ (kcal/mol)	-19 ± 59	24 ± 26	-234 ± 79	-91 ± 55	-179 ± 64	-48 ± 57
$E_{non-bonded}$ (kcal/mol)	-252 ± 49	-181 ± 46	-319 ± 51	-225 ± 53	-32 ± 83	-134 ± 66
$E_{van\ der\ Waals}$ (kcal/mol)	-31 ± 7	-34 ± 7	-41 ± 7	-37 ± 7	-23 ± 5	-28 ± 4
$E_{electrostatic}$ (kcal/mol)	-221 ± 52	-147 ± 48	-278 ± 49	-187 ± 49	-298 ± 84	-106 ± 65
$E_{ambiguous\ intermolecular\ restraints}$ (kcal/mol)	233 ± 34	205 ± 31	85 ± 56	134 ± 40	142 ± 52	-87 ± 45
No. of AIR violations	3.6 ± 0.5	2.9 ± 0.7	2.4 ± 1.2	3.3 ± 1.0	3 ± 1.2	2.2 ± 1.1

[a]The ambiguous interaction restraints (AIR) was based on NMR CSP data and mutagenesis studies.

[b]The number for Pgp2 corresponds to the amino acid number in the native sequence. The number 1, 2, and 3 for the murotetrapeptide corresponds to the residues GlcNAc, MurNAc and tetrapeptide.

[c]In each cluster, the energetic statistics were calculated over the 10 structures with the best HADDOCK scores.

Table S3. Strains and primers used in this study.

Strain	Description
E. coli BL21-Pgp2	*E. coli* BL21 (λDE3) strain harboring plasmid pET15b-Pgp2$^{43\text{-}325}$
E. coli BL21-Pgp2^{K307A}	*E. coli* BL21 (λDE3) strain harboring Pgp2^{K307A} expression plasmid
C. jejuni 81-176	An isolate from a raw-milk diarrhea outbreak [a]
Δ*pgp2*	*C. jejuni* strain 81-176 with a pgp2 deletion [a]
Δ*pgp2*^{C}	Strain Δ*pgp2* complemented with a copy of pgp2 integrated at the rRNA spacer region found in *C. jejuni* [a]
H157A	Complement of strain Δ*pgp2* with catalytically inactive SDM variant
C174S	Complement of strain Δ*pgp2* with catalytically inactive SDM variant
Y233A	Complement of strain Δ*pgp2* with SDM variant in the NTF2 domain
F242A	Complement of strain Δ*pgp2* with SDM variant in the NTF2 domain
K257A	Complement of strain Δ*pgp2* with SDM variant in the NTF2 domain
K307A	Complement of strain Δ*pgp2* with SDM variant in the NTF2 domain
E324Q	Complement of strain Δ*pgp2* with SDM variant in the NTF2 domain
**Primer ID**	**Primer Sequence 5′ to 3′**
Protein expression vector	
Pgp2	(F) GGGAATTC*CATATG*CAAAAGGATTTTTGGTTAAGT[b]<br>(R) CCG*CTCGAG*TTATTGCTCTGCTAAAATTTT[b]
Pgp2^{K307A}	(F) CAGTTTAGAGGCGAT**GCA**ATTTTATACGTTAAG[b]<br>(R) CTTAACGTATAAAAT**TGC**ATCGCCTCTAAACTG[b]
Chromosome sequencing	
Spe1	GCGACTAGTTGCCTAAAAAAGACATTCCTATAAA
198R	TTAATAAAGCAAGTCGTTTTAACAA
554F	AGGCCGTAAGTTTAATCCAGGTGA
cat-2	GTTTTTTGGATGAATTACAAGA
Site-directed mutagenesis	
C174S	(F) TAAAACAAGAGGA**AGT**ATTGCTTTATT[b]<br>(R) AATAAAGCAATA**ACT**TCCTCTTGTTTTA[b]
H157A	(F) GTGGTATTTGGATC**GCT**GGCTATCCTTTAG[b]<br>(R) CTAAAGGATAGCC**AGC**GATCCAAATACCAC[b]
Y233A	(F) GTGACACTAATACC**GCT**TTAAGTTTTTATG[b]<br>(R) CATAAAAACTTAA**AGC**GGTATTAGTGTCAC[b]
F242A	(F) GATGAGCAAGAA**GCT**AAACGTTTTGAT[b]<br>(R) ATCAAAACGTTT**AGC**TTCTTGCTCATC[b]
K257A	(F) TTTGCTTCCATG**GCA**AAATCTATTTT[b]<br>(R) AAAATAGATTT**TGC**CATGGAAGCAAA[b]
K307A	(F) CAGTTTAGAGGCGAT**GCA**ATTTTATACGTTAAG[b]<br>(R) CTTAACGTATAAAAT**TGC**ATCGCCTCTAAACTG[b]
E324Q	(F) GAAAATTTTAGCA**CAG**CAATAAATGTC[b]<br>(R) GACATTTATTG**CTG**TGCTAAAATTTTC[b]

[a]Source of strains are as previously described (12).

[b]Restriction enzyme digestion sites are in italics. The three nucleotides of the variant residue are in bold. The nucleotide that is different to the native sequence is underlined.

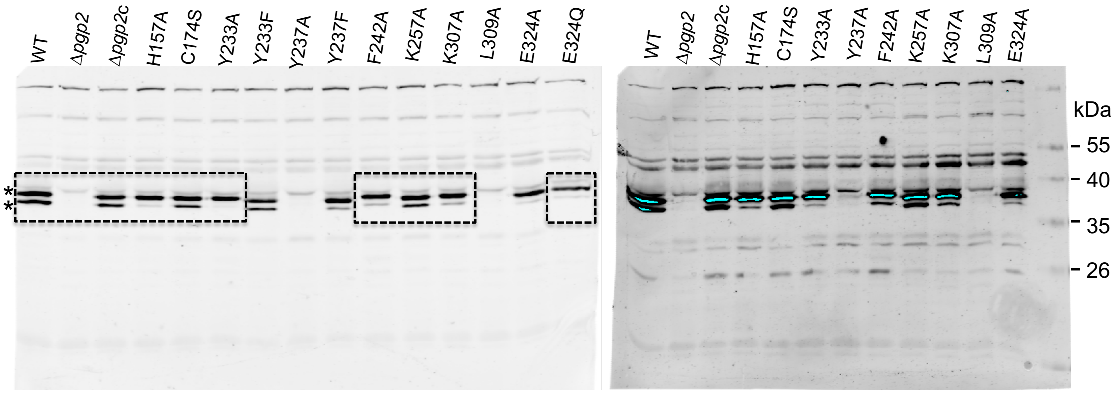

Western blot analysis of *C. jejuni* whole cell lysate against anti-Pgp2 antibody

Figure S1. Western blots of whole cell lysates of *C. jejuni* strains probed with anti-Pgp2 polyclonal antibodies. Strains are labeled at the top of each lane. A protein molecular weight reference ladder is shown on the right. The bands corresponding to Pgp2 are labelled with '*'. The dash line boxes indicate the regions presented in Figure 2C. *C. jejuni* cells at log phase were lysed using 0.1% Triton X-100. Lysates were quantified by total protein at OD_{595} using Bradford reagent (Bio-Rad). The lysates were separated on a 12% SDS-PAGE gel followed by transferred onto nitrocellulose membrane before antibody incubation. The primary antibody is rabbit anti-Pgp2. The secondary antibody is conjugated to Alexa Fluor 680 (Invitrogen). The gel was imaged at 700 nm by an Odyssey Classic Imaging System (LI-COR). The bright cyan lanes indicated signal saturation.

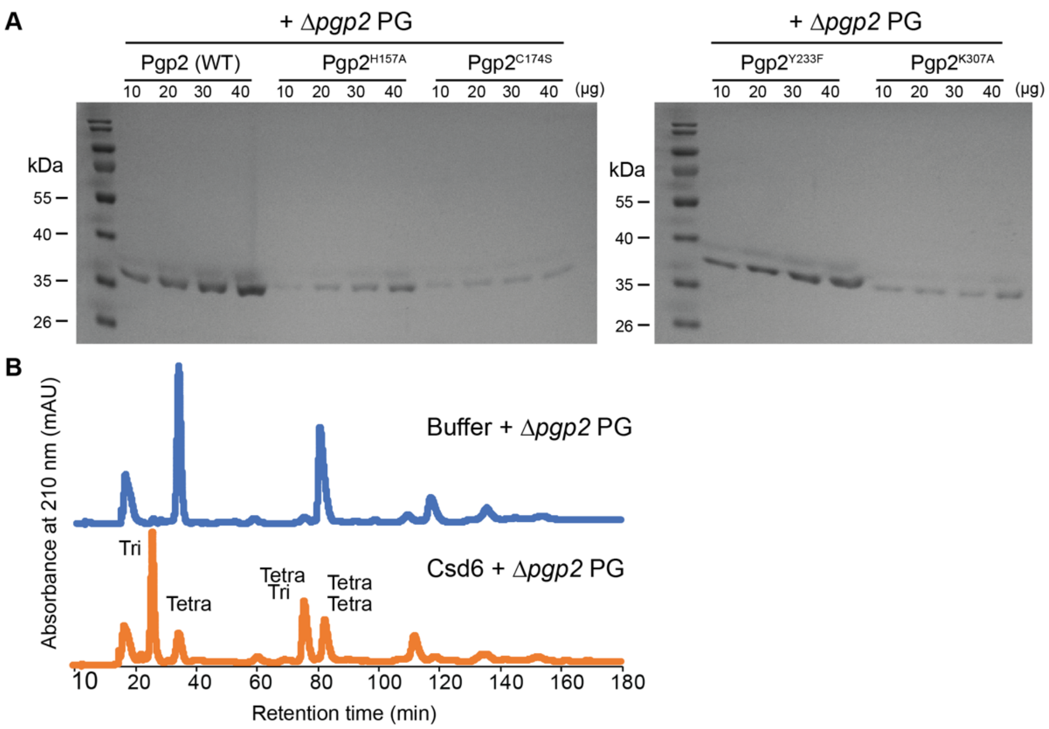

Figure S2. Binding of Pgp2 mutants to Δ*pgp2* PG and digestion by Csd6. (A) Pgp2 and variant proteins pulled-down by PG isolated from *C. jejuni* Δ*pgp2*. Pgp2 proteins at the indicated amounts were incubated with purified PG (50 µg) in a 250 µL reaction at 4 °C for 30 min, followed by centrifugation at 13,000 rpm for 10 min. Insoluble PG and pulled-down proteins were washed 3 times with 1 mL of buffer (50 mM Tris pH 7.0 and 150 mM NaCl) to remove unbound proteins. Insoluble PG and pulled-down proteins were analyzed by SDS-PAGE stained with Coomassie Blue. (B) HPLC muropeptide profile of purified *C. jejuni* Δ*pgp2* PG after digestion with Csd6. Samples were separated on a C_{18} column using a gradient from 100% buffer A (50 mM sodium phosphate pH 4.3) to 100% buffer B (50 mM sodium phosphate pH 4.9 and methanol 15% (v/v)) over 200 minutes.

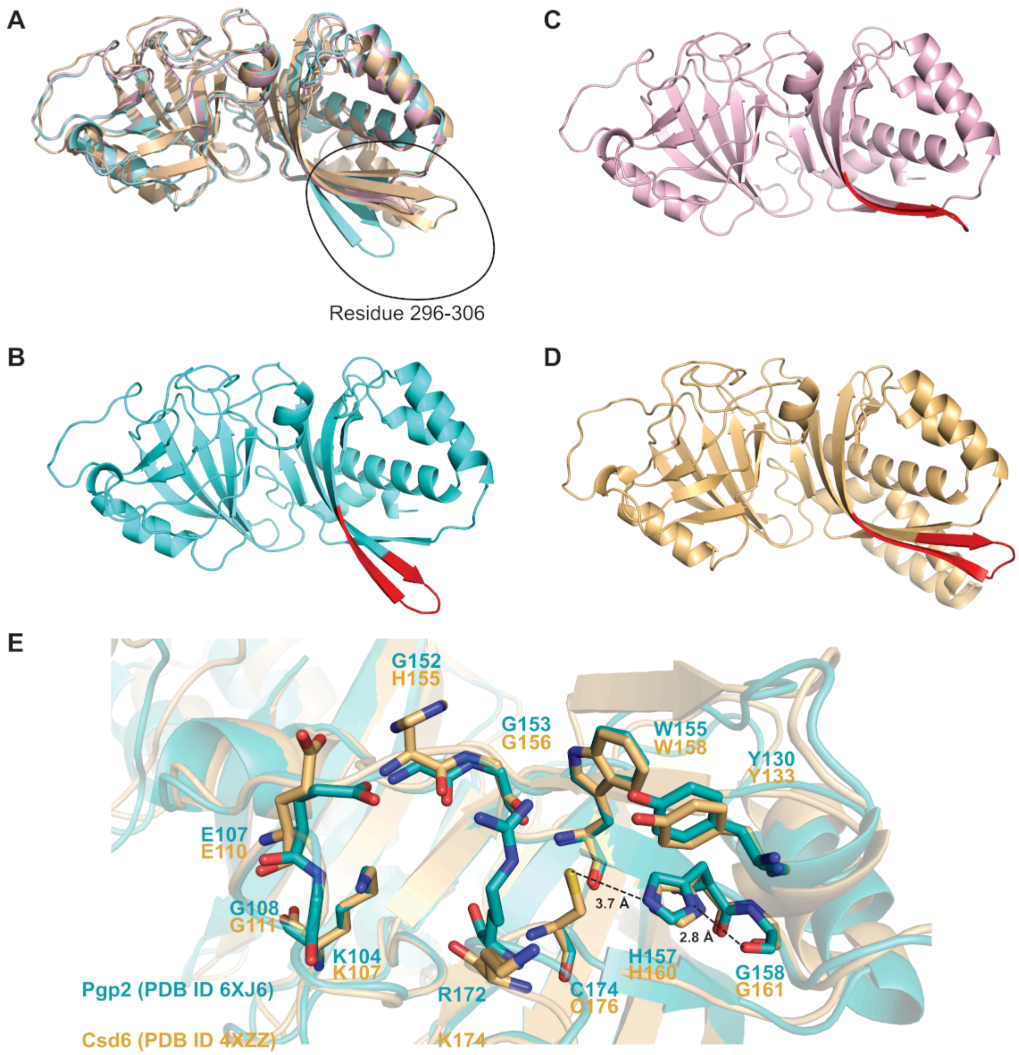

Figure S3. Superimpositions of the Pgp2, Pgp2^{K307A} and Csd6 structures. (A) Cartoon representation of an overlay of Pgp2 (cyan), Pgp2^{K307A} monomer B (pink) and Csd6 monomer A (orange, PDB ID: 4XZZ). Cartoon representation of Pgp2 (B), Pgp2^{K307A} (C), and Csd6 (D) structures. Residues 296-306 of strands β9-β10 form a flexible lip (circled or highlighted in red). (E) Superimposition of the active sites of Pgp2 (PDB ID: 6XJ6) and Csd6 (PDB ID: 4XZZ). Residues of the catalytic triad and substrate binding site are shown in stick form (nitrogen, blue; oxygen, red; sulfur, yellow). A hydrogen bond network between residues of the triad is drawn as dashed lines.

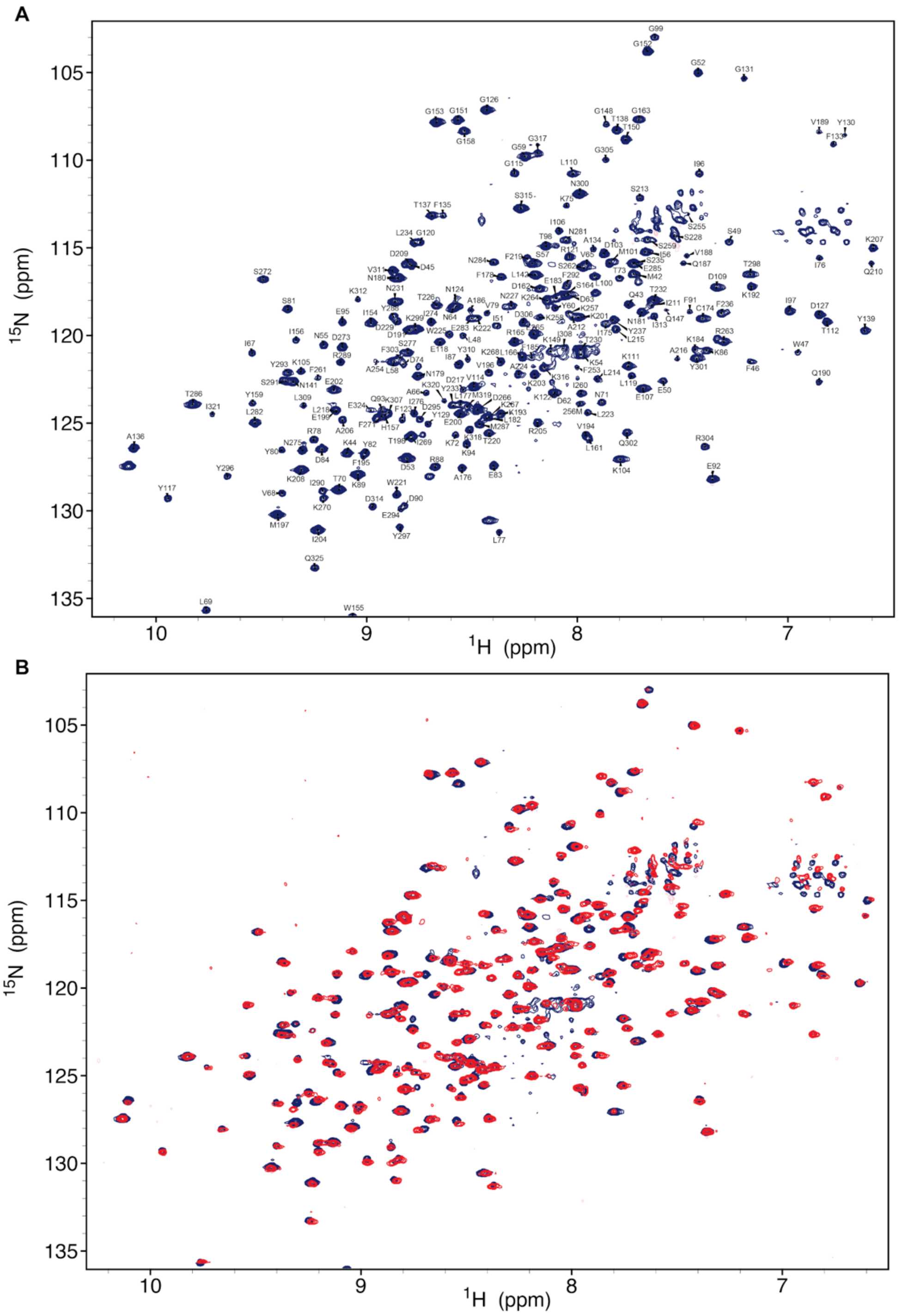

Figure S4. HSQC spectra of Pgp2 assignment of backbone amide ^{15}N and ^{1}H^{N} cross resonances. (A) Assigned ^{15}N-BEST-TROSY-HSQC spectrum of a ^{2}H-^{13}C-^{15}N labeled Pgp2 sample, recorded at 25 °C with an 850 MHz NMR spectrometer. Based on 3D ^{1}H-^{13}C-^{15}N scalar correlation experiments, backbone amide ^{1}H^{N}-^{15}N signals were automatically assigned using PINE, followed by manual verification with NMRFAM-SPARKY. (B) Overlaid ^{15}N-BEST-TROSY-HSQC spectra of Pgp2 uniformly labeled with only ^{15}N (red)

and with ^{2}H, ^{13}C and ^{15}N (blue). Although deuteration was required to obtain assignments, ^{15}N-labeling was sufficient for NMR-monitored titrations.

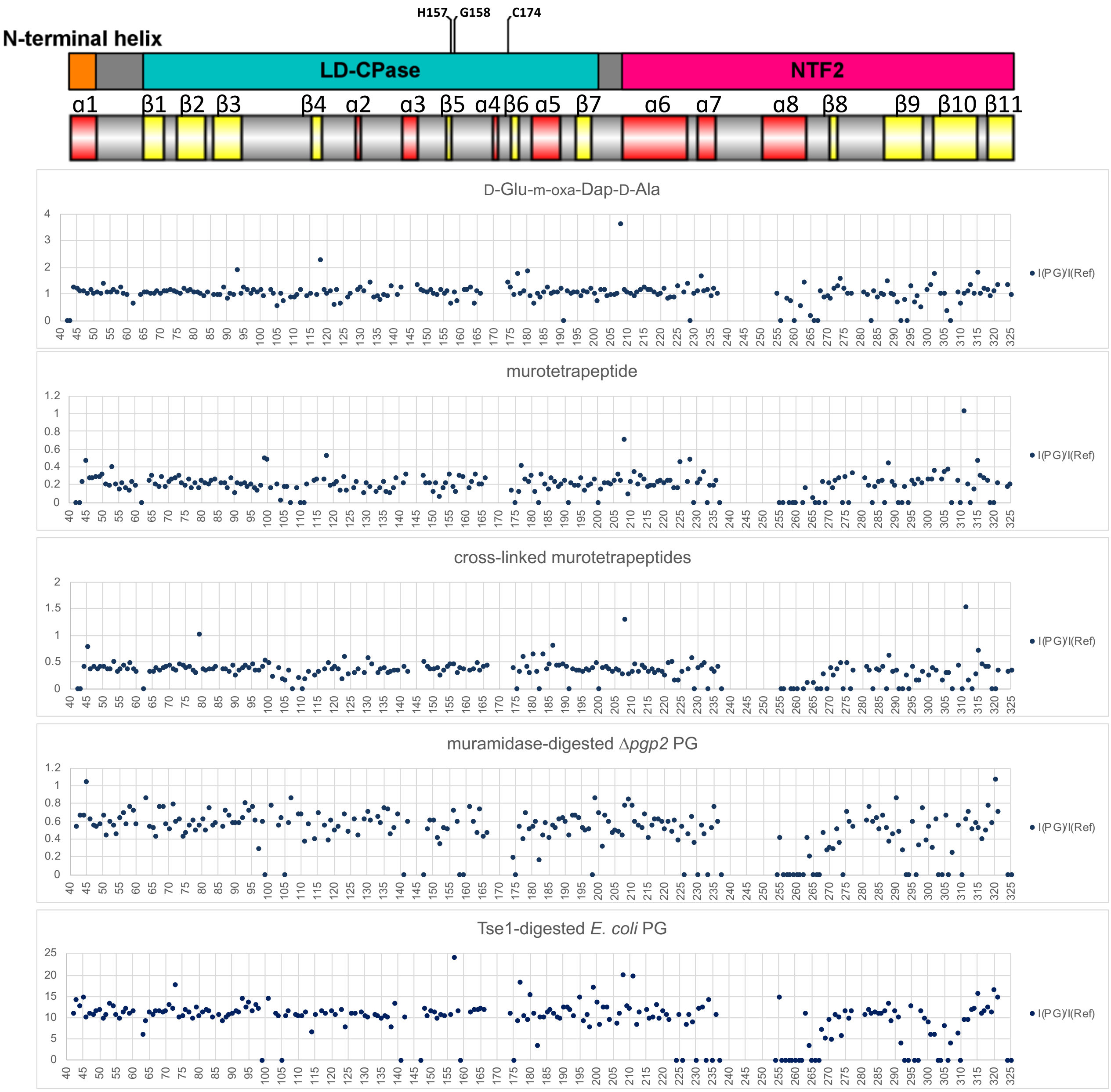

Figure S5. Relative intensity analysis of ^{15}N-labeled Pgp2 interacting with a panel of PG derived ligands. Relative intensity is calculated as I(PG)/I(Ref), where I(Ref) and I(PG) denote the height of an amide ^{1}H^{N}-^{15}N peak in ^{15}N-BEST-TROSY spectra recorded in the absence versus the presence of a PG ligand at the endpoint (highest) ligand:protein ratio for each given titration experiment. Signal intensities were not corrected for dilution (~0.99 with D-Glu-m-oxa-Dap-D-Ala, ~0.9 with the murotetrapeptide, cross-linked

murotetrapeptides and muramidase-digested PG, and ~0.5 for the Tse1-digested *E. coli* PG) or in the case of Tse1-digested *E. coli* PG, increased acquisition time to better detect weaker signals.

Supplemental References

1. Soni AS, Lin CS, Murphy MEP, Tanner ME. 2019. Peptides Containing meso-Oxa-Diaminopimelic Acid as Substrates for the Cell-Shape-Determining Proteases Csd6 and Pgp2. ChemBioChem 20:1591-1598.
2. Cai M, Huang Y, Yang R, Craigie R, Clore GM. 2016. A simple and robust protocol for high-yield expression of perdeuterated proteins in Escherichia coli grown in shaker flasks. J Biomol NMR 66:85-91.
3. Otwinowski Z, Minor W. 1997. Processing of X-ray diffraction data collected in oscillation mode. Methods Enzymol 276:307-26.
4. Liebschner D, Afonine PV, Baker ML, Bunkoczi G, Chen VB, Croll TI, Hintze B, Hung LW, Jain S, McCoy AJ, Moriarty NW, Oeffner RD, Poon BK, Prisant MG, Read RJ, Richardson JS, Richardson DC, Sammito MD, Sobolev OV, Stockwell DH, Terwilliger TC, Urzhumtsev AG, Videau LL, Williams CJ, Adams PD. 2019. Macromolecular structure determination using X-rays, neutrons and electrons: recent developments in Phenix. Acta Crystallogr D Struct Biol 75:861-877.
5. Emsley P, Lohkamp B, Scott WG, Cowtan K. 2010. Features and development of Coot. Acta Crystallogr D Biol Crystallogr 66:486-501.
6. Kabsch W. 2010. Xds. Acta Crystallogr D Biol Crystallogr 66:125-32.
7. Winn MD, Ballard CC, Cowtan KD, Dodson EJ, Emsley P, Evans PR, Keegan RM, Krissinel EB, Leslie AG, McCoy A, McNicholas SJ, Murshudov GN, Pannu NS, Potterton EA, Powell HR, Read RJ, Vagin A, Wilson KS. 2011. Overview of the CCP4 suite and current developments. Acta Crystallogr D Biol Crystallogr 67:235-42.
8. Lescop E, Schanda P, Brutscher B. 2007. A set of BEST triple-resonance experiments for time-optimized protein resonance assignment. J Magn Reson 187:163-9.
9. Delaglio F, Grzesiek S, Vuister GW, Zhu G, Pfeifer J, Bax A. 1995. NMRPipe: a multidimensional spectral processing system based on UNIX pipes. J Biomol NMR 6:277-93.
10. Lee W, Tonelli M, Markley JL. 2015. NMRFAM-SPARKY: enhanced software for biomolecular NMR spectroscopy. Bioinformatics 31:1325-7.
11. Bahrami A, Assadi AH, Markley JL, Eghbalnia HR. 2009. Probabilistic interaction network of evidence algorithm and its application to complete labeling of peak lists from protein NMR spectroscopy. PLoS Comput Biol 5:e1000307.
12. Frirdich E, Vermeulen J, Biboy J, Soares F, Taveirne ME, Johnson JG, DiRita VJ, Girardin SE, Vollmer W, Gaynor EC. 2014. Peptidoglycan LD-carboxypeptidase Pgp2 influences Campylobacter jejuni helical cell shape and pathogenic properties and provides the substrate for the DL-carboxypeptidase Pgp1. J Biol Chem 289:8007-18.
